# Supplementary material for: Lactate dehydrogenase A regulates tumor-macrophage symbiosis to promote glioblastoma progression
Source: Nat Commun. 2024 Mar 5;15:1987. doi: 10.1038/s41467-024-46193-z (PMC10914854; doi:10.1038/s41467-024-46193-z)
Supplement: Supplementary file 1 — Supplementary Information [file 41467_2024_46193_MOESM1_ESM.pdf]

Supplementary Information Fig. 1

a

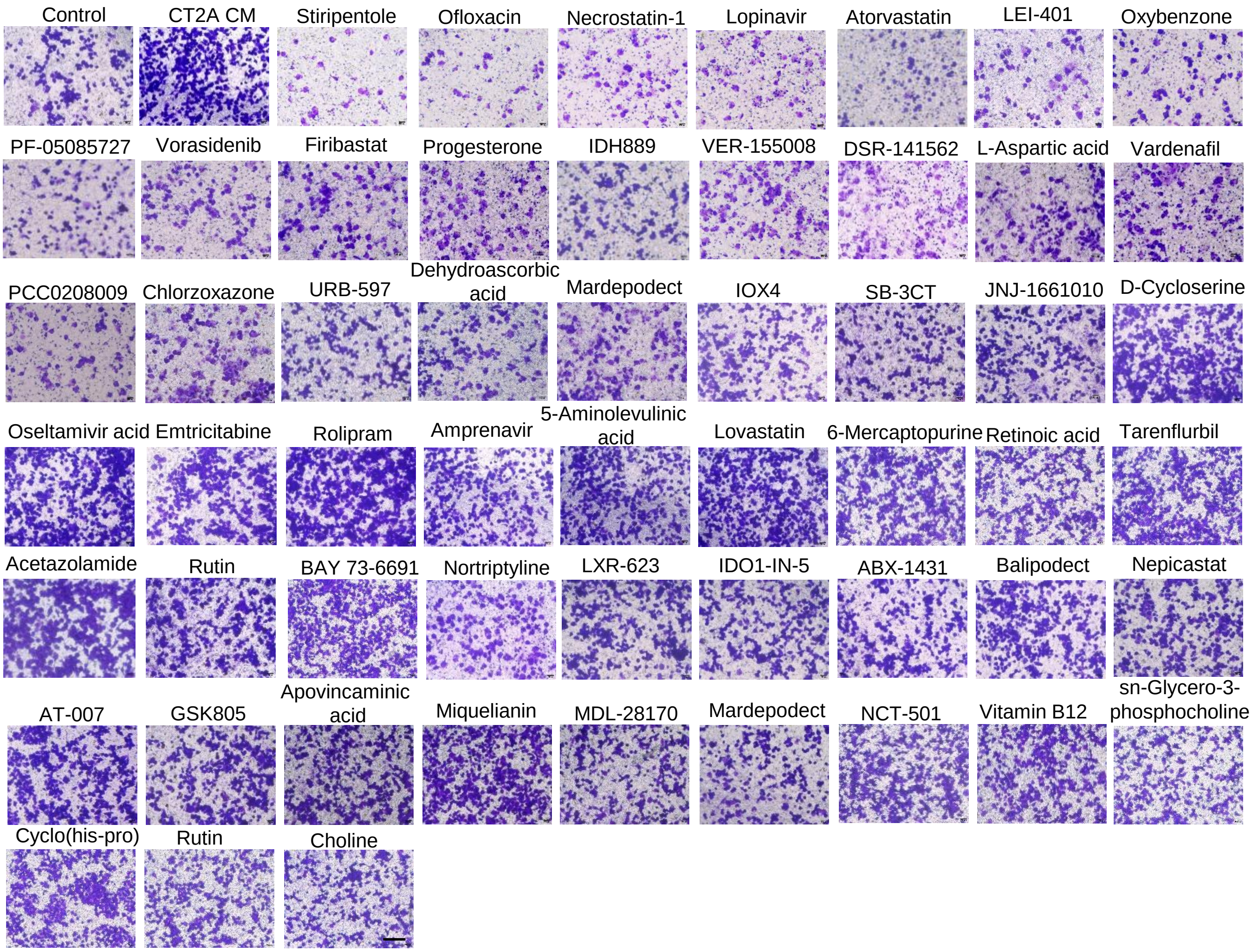

b

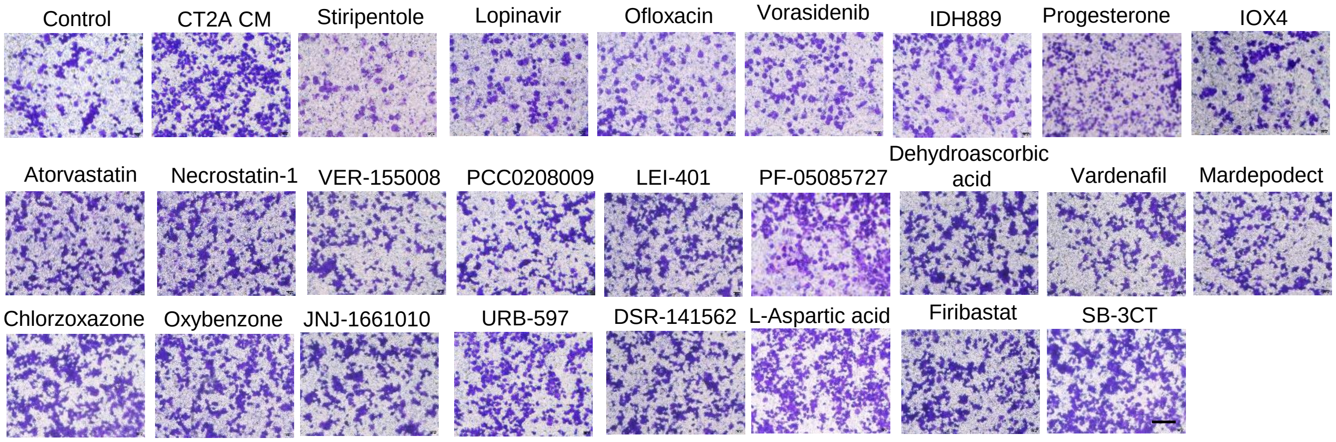

Supplementary Fig. S1. Screening of metabolic compounds that can inhibit CT2A cell-induced macrophage migration. (a) Representative images of the migration of Raw264.7 macrophages following stimulation with conditioned media (CM) from CT2A cells treated with or without a cluster of 55 brain-penetrant small-molecule compounds with metabolic reprogramming functions at 10 μM. Scale bar, 100 μm. **(b)** Representative images of the migration of Raw264.7 macrophages following stimulation with conditioned media (CM) from CT2A cells treated with or without a cluster of 24 brain-penetrant small-molecule compounds with metabolic reprogramming functions at 5 μM. Scale bar, 100 μm. A representative example of three replicates is shown for (a and b).

Supplementary Information Fig. 2

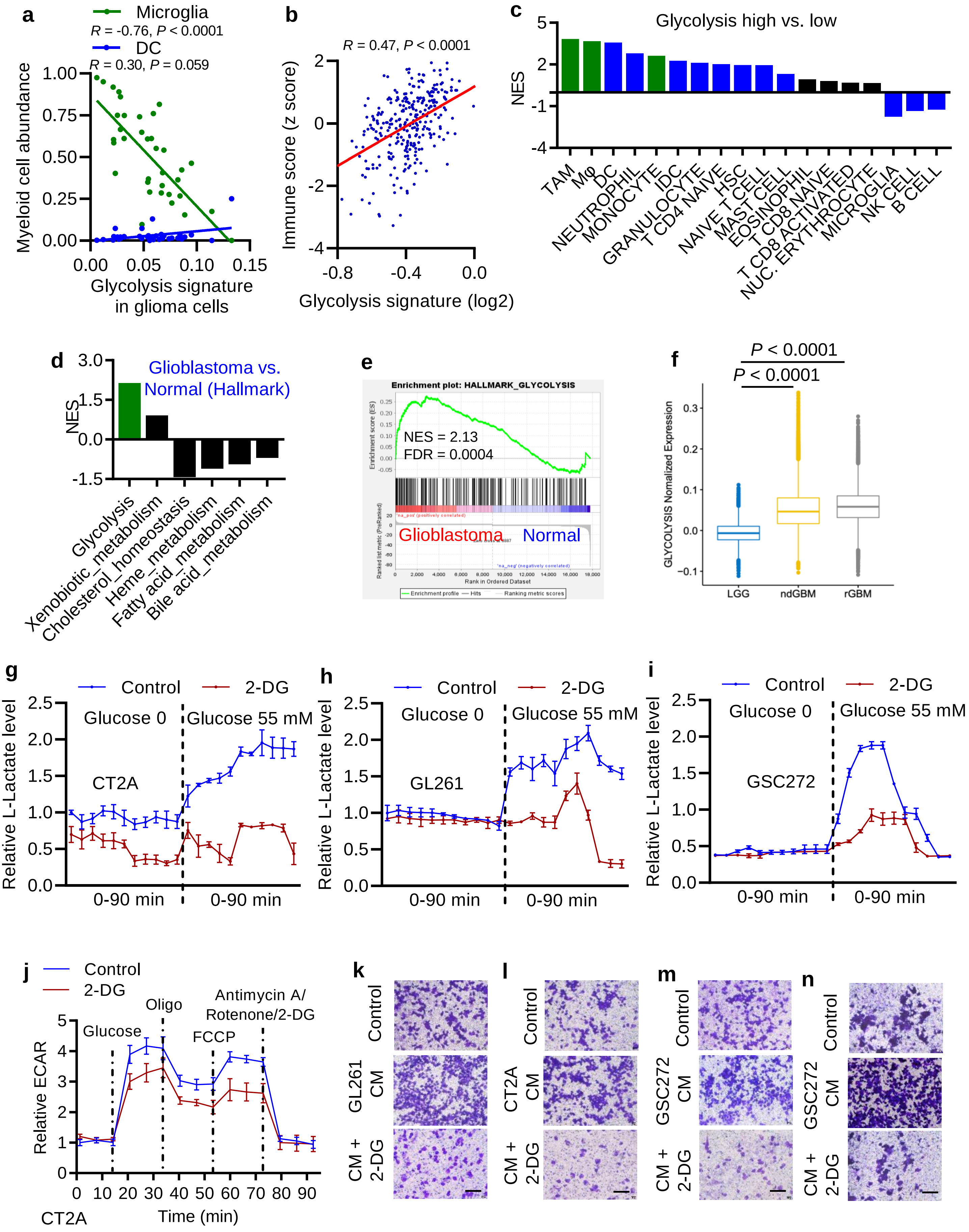

Supplementary Fig. S2. The role of glioblastoma cell glycolysis in regulating immune response and macrophage infiltration. (a) The correlation analysis between glycolysis signature in glioblastoma cells and the abundance of microglia, and dendritic cells (DCs) in glioblastoma patient tumors based on single-cell RNA sequencing data [34]. *R* and *P* values are shown. **(b)** The correlation analysis between glycolysis signature and immune score in TCGA glioblastoma dataset (n = 300). The immune score was determined based on expression data from TCGA glioblastoma dataset [31]. *R* and *P* values are shown. **(c)** GSEA analysis for distinct types of immune cells in glycolysis signature-high (n = 119) and -low (n = 119) patients in TCGA glioblastoma dataset. Green bars indicate macrophage-related signatures, green and blue bars indicate the signatures that are enriched in glycolysis signature-high tumors (FDR<0.25). **(d)** GSEA analysis for distinct types of metabolic signatures in glioblastoma patient tumors (n = 478) versus normal brains (n = 10) from the TCGA glioblastoma dataset. Green bar indicates the glycolysis signature that is significantly enriched in glioblastoma (FDR<0.25). **(e)** GSEA for glycolysis signature in glioblastoma patient tumors (n = 478) versus normal brains (n = 10) from the TCGA glioblastoma dataset. NES and FDR values are shown. **(f)** Expression of glycolysis signature in glioma cells of low-grade gliomas (LGG), newly diagnosed glioblastoma (ndGBM), and recurrent glioblastoma (rGBM) based on single-cell RNA sequencing data [34]. **(g-i)** Glycolytic activity assay on CT2A cells (**g**), GL261 cells (**h**), and GSC272 (**i**) treated with or without glycolysis inhibitor 2-deoxy-D-glucose (2-DG, 10 mM) in the presence or absence of glucose (55 mM). n = 3 independent samples. **(j)** Extracellular acidification rate (ECAR) of CT2A cells treated with or without 2-DG (10 mM). ECAR was obtained from the Seahorse experiments and glucose was added at indicated time point. n = 6 independent samples. **(k, l)** Representative images of the migration of Raw264.7 macrophages (**k**) and primary mouse bone marrow-derived macrophages (BMDMs, **l**) following stimulation with conditioned media (CM) from GL261 and CT2A cells, respectively, treated with or without 2-DG (10 mM). Scale bar, 100 μm. **(m, n)** Representative images of the migration of THP-1 macrophages (**m**) and primary human BMDMs (**n**) following stimulation with CM from GSC272 treated with or without 2-DG (10 mM). Scale bar, 100 μm. A representative example of five replicates is shown for (k–n). The experiments for (j) were independently repeated at least three times. Statistical analyses were determined by Pearson's correlation test (a, b) and one-way ANOVA test (f). Source data are provided as a Source Data file.

Supplementary Information Fig. 3

a

Genes	Survival	Immune score	Mφ signature
HK1	X	*R*=0.177; *P*=0.002	*R*=0.289; *P*<0.0001
HK2	X	*R*=0.117; *P*=0.043	*R*=0.185; *P*<0.0001
HK3	X	*R*=0.541; *P*<0.0001	*R*=0.702; *P*<0.0001
PGM1	√	*R*=-0.130; *P*=0.024	*R*=-0.044; *P*=0.331
PGM2	X	*R*=0.251; *P*<0.0001	*R*=0.352; *P*<0.0001
LDHA	√	***R*=0.277**; ***P*<0.0001**	***R*=0.407**; ***P*<0.0001**
LDHB	√	*R*=-0.207; *P*=0.0003	*R*=-0.282; *P*<0.0001
MDH1	√	*R*=0.09; *P*=0.143	*R*=0.055; *P*=0.222
MDH2	X	*R*=-0.014; *P*=0.804	*R*=0.0242; *P*=0.594
FH	X	*R*=-0.023; *P*=0.691	*R*=-0.123; *P*=0.0065
SDHA	X	*R*=-0.089; *P*=0.123	*R*=-0.140; *P*=0.002
SUCLA2	X	*R*=-0.031; *P*=0.594	*R*=-0.072; *P*=0.1136
OGDH	X	*R*=0.122; *P*=0.034	*R*=0.108; *P*=0.0174
IDH3A	X	*R*=0.186; *P*=0.0012	*R*=0.163; *P*=0.0003
IDH3B	X	*R*=-0.182; *P*=0.0016	*R*=-0.252; *P*<0.0001
IDH3G	X	*R*=-0.009; *P*=0.874	*R*=-0.085; *P*=0.06
CS	X	*R*=0.014; *P*=0.813	*R*=-0.047; *P*=0.299
ACO1	X	*R*=-0.051; *P*=0.378	*R*=-0.025; *P*=0.582

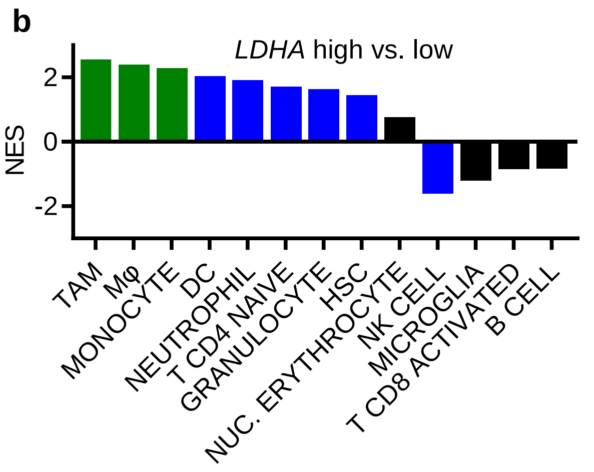

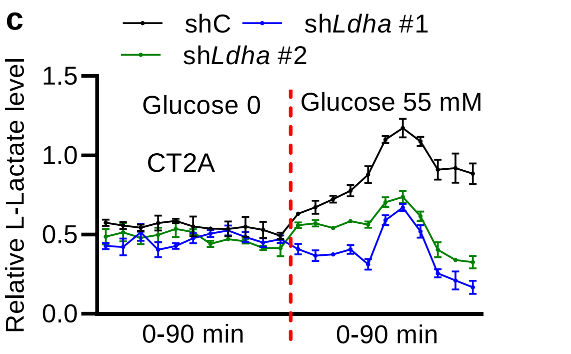

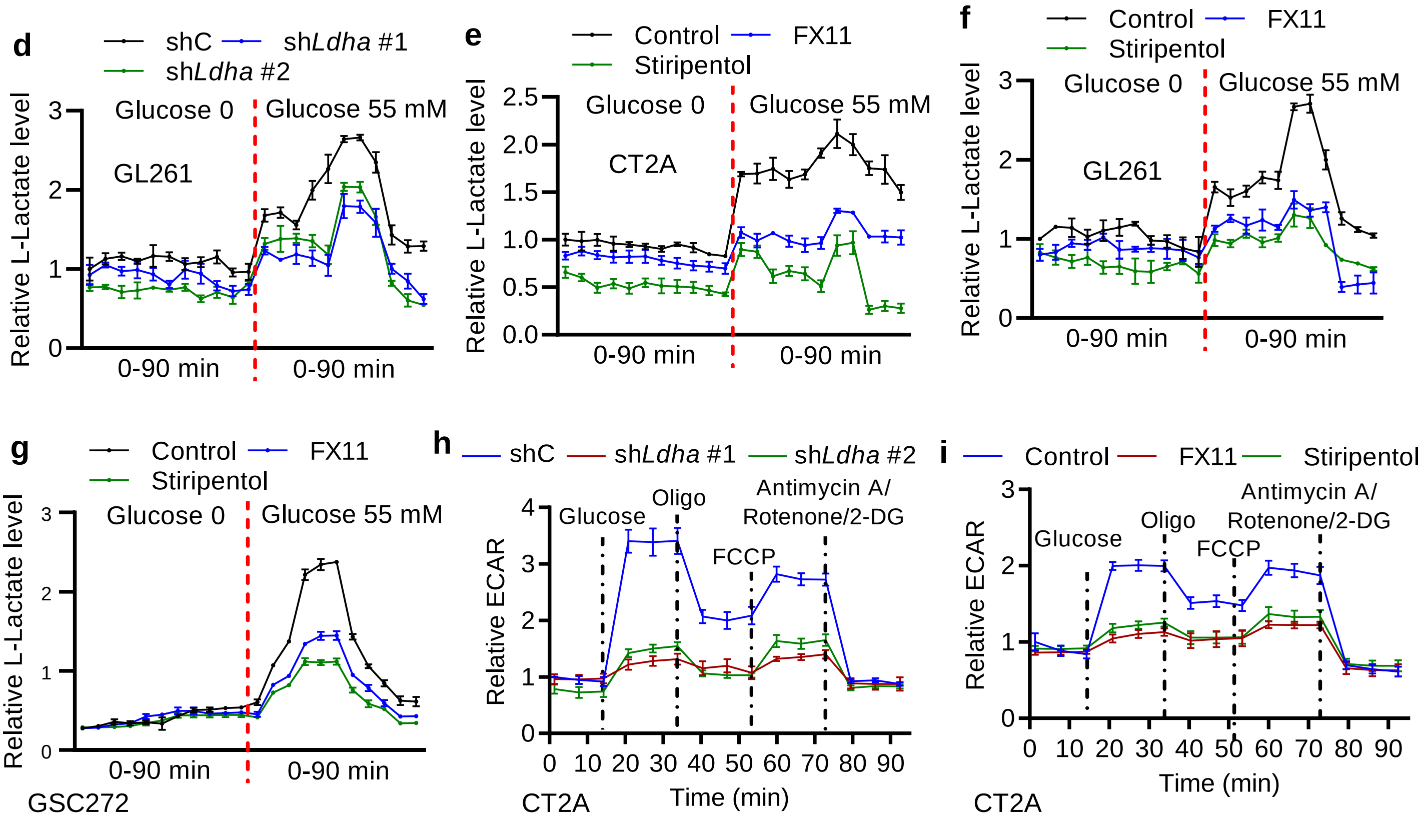

Supplementary Fig. S3. LDHA regulates glioblastoma cell glycolysis and correlates with macrophage signature in glioblastoma patients. (a) The correlation analysis between key glycolysis and TCA enzymes (e.g., HK1, HK2, HK3, PGM1, PGM2, LDHA, LDHB, MDH1, MDH2, FH, SDHA, SUCLA2, OGDH, IDH3A, IDH3B, IDH3G, CS, and ACO1) with patient survival, immune score, and macrophage signature in TCGA glioblastoma dataset (n = 478). Pearson's correlation test and log-rank test were used for correlation and survival analysis, respectively. X and √ indicate no and negative correlation with survival, respectively. *R* and *P* values are shown. Blue and red colors indicate positive and negative correlation, respectively. **(b)** GSEA analysis for distinct types of immune cells *LDHA*-high (n = 119) and -low (n = 119) patients in TCGA glioblastoma dataset. **(c, d)** Glycolytic activity assay on CT2A (**c**) and GL261 (**d**) cells expressing shRNA control (shC) and *Ldha* shRNAs (sh*Ldha*) in the presence or absence of glucose (55 mM). n = 3 independent samples. **(e-g)** Glycolytic activity assay on CT2A cells (**e**), GL261 cells (**f**) and GSC272 (**g**) treated with or without LDHA inhibitor FX11 (8 μM) or stiripentol (10 μM) in the presence or absence of glucose (55 mM). n = 3 independent samples. **(h)** Extracellular acidification rate (ECAR) of CT2A cells expressing shC and sh*Ldha*. ECAR was obtained from the Seahorse experiments and glucose was added at indicated time point. n = 6 independent samples. **(i)** ECAR of CT2A cells treated with or without LDHA inhibitor FX11 (8 μM) or stiripentol (10 μM). ECAR was obtained from the Seahorse experiments and glucose was added at indicated time point. n = 6 independent samples. The experiments for (h and i) were independently repeated at least three times. Statistical analyses were determined by Pearson's correlation test and log-rank test (a). Source data are provided as a Source Data file.

Supplementary Information Fig. 4

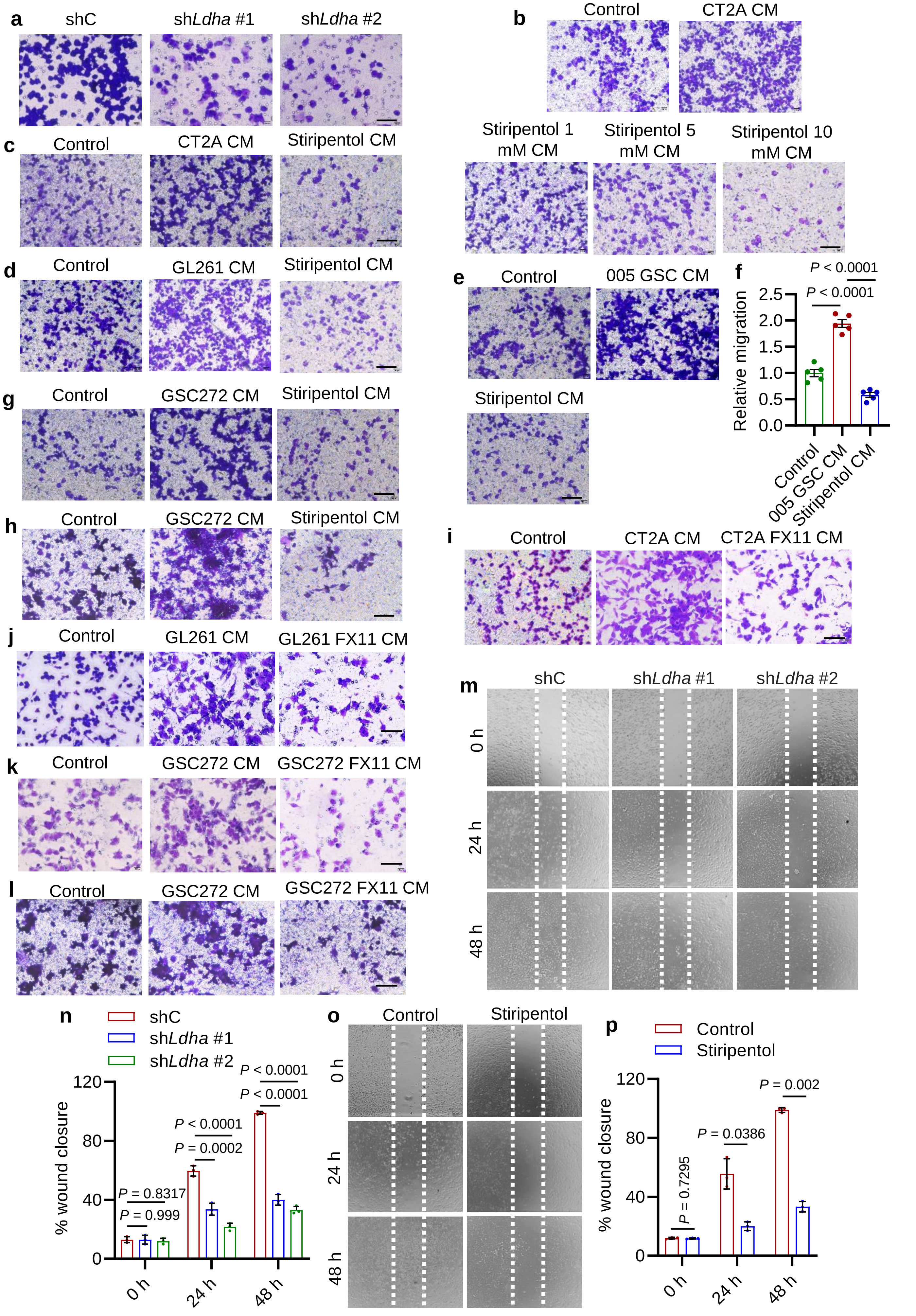

Supplementary Fig. S4. Glioblastoma cell LDHA inhibition impairs macrophage migration. (a) Representative images of the migration of Raw264.7 macrophages from a transwell analysis following stimulation with CM from GL261 cells expressing shRNA control (shC) and *Ldha* shRNAs (sh*Ldha*). Scale bar, 100 μm. **(b)** Representative images of the migration of Raw264.7 macrophages from a transwell analysis following stimulation with CM from CT2A cells treated with or without stiripentol at indicated concentrations. Scale bar, 100 μm. **(c, d)** Representative images of the migration of primary mouse bone marrow-derived macrophages (BMDMs) and Raw264.7 macrophages from a transwell analysis following stimulation with CM from CT2A (**c**) and GL261 (**d**) cells, respectively, treated with or without stiripentol (10 μM). Scale bar, 100 μm. **(e, f)** Representative images (**e**) and quantification (**f**) of migration of Raw264.7 macrophages from a transwell analysis following stimulation with CM from 005 GSCs treated with or without stiripentol (10 μM). Scale bar, 100 μm. n = 5 independent samples. **(g, h)** Representative images of the migration of THP-1 macrophages (**g**) and primary human BMDMs (**h**) from a transwell analysis following stimulation with CM from GSC272 treated with or without stiripentol (10 μM). Scale bar, 100 μm. **(i, j)** Representative images of the migration of Raw264.7 macrophages from a transwell analysis following stimulation with CM from CT2A (**i**) or GL261 (**j**) cells treated with or without FX11 (8 μM). Scale bar, 100 μm. **(k, l)** Representative images of the migration of THP-1 macrophages (**k**) and primary human BMDMs (**l**) from a transwell analysis following stimulation with CM from GSC272 treated with or without FX11 (8 μM). Scale bar, 100 μm. **(m, n)** Representative images (**m**) and quantification (**n**) of relative wound healing migration of Raw264.7 macrophages from a scratch assay analysis following stimulation with CM from CT2A cells expressing shC and sh*Ldha*. n = 3 independent samples. **(o, p)** Representative images (**o**) and quantification (**p**) of relative wound healing migration of Raw264.7 macrophages from a scratch assay analysis following stimulation with CM from CT2A cells treated with or without stiripentol (10 μM) n = 3 independent samples. A representative example of five and three replicates is shown for (a-e and h-l) and (m and o), respectively. The experiments were independently repeated at least two times. Data from multiple replicates are presented as mean ± SEM. Statistical analyses were determined by one-way ANOVA test (f, n) and Student's t-test (p). Source data are provided as a Source Data file.

Supplementary Information Fig. 5

a

Genes	Survival	Mφ signature
CCL2	√	*R*=0.718; *P*<0.0001
CCL7	√	*R*=0.683; *P*<0.0001
IL1B	X	*R*=0.569; *P*<0.0001
IL1RAP	√	*R*=0.387; *P*<0.0001
IL1RN	X	*R*=0.576; *P*<0.0001
MMP9	X	*R*=0.385; *P*<0.0001
NPY	X	*R*=-0.032; *P*=0.477
PLAU	√	*R*=0.624; *P*<0.0001
PROS1	X	*R*=0.537; *P*<0.0001
S100A8	√	*R*=0.734; *P*<0.0001
SLPI	X	*R*=0.509; *P*<0.0001

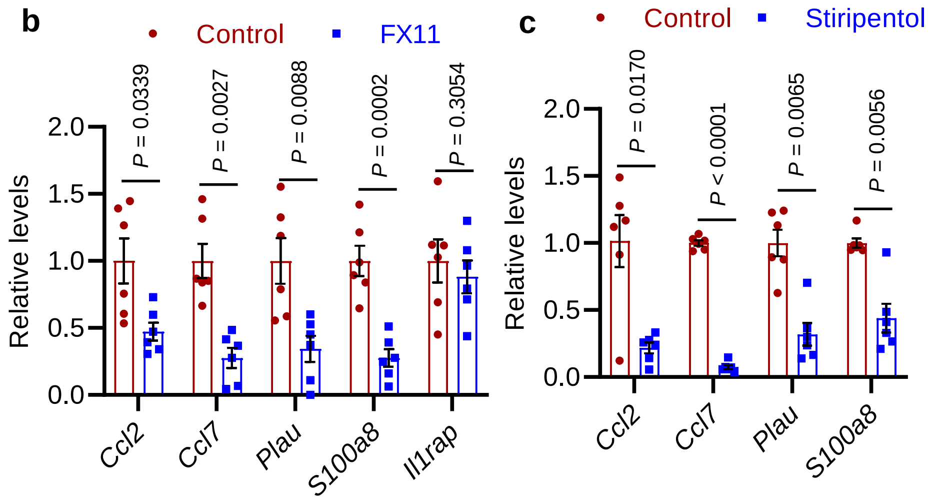

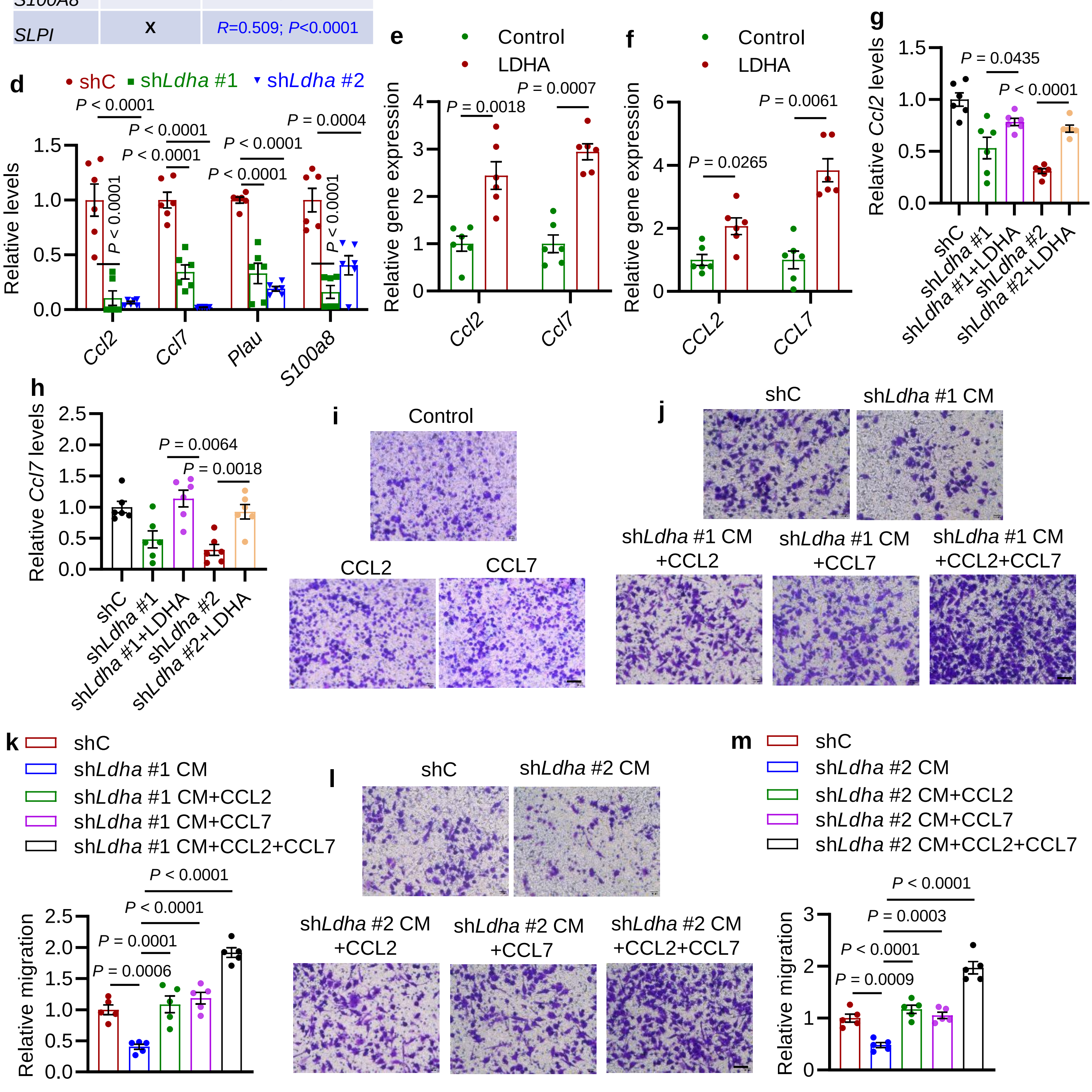

Supplementary Fig. S5. CCL2 and CCL7 are the key chemokines responsible for LDHA-induced macrophage migration. (a) The correlation analysis between cytokines (e.g., *CCL2*, *CCL7*, *IL1B*, *IL1RAP*, *IL1RN*, *MMP9*, *NPY*, *PLAU*, *PROS1*, *S100A8* and *SLPI*) with patient survival and macrophage signature in TCGA glioblastoma dataset (n = 478). Pearson's correlation test and log-rank test were used for correlation and survival analysis, respectively. X and √ indicate no and negative correlation with survival, respectively. *R* and *P* values are shown. Blue colors indicate positive correlation. **(b)** RT-qPCR for *Ccl2*, *Ccl7*, *Il1rap*, *Plau*, and *S100a8* in control and FX11-treated GL261 cells. The values were expressed as the fold change. n = 6 independent samples. **(c)** RT-qPCR for *Ccl2*, *Ccl7*, *Plau*, and *S100a8* in control and stiripentol-treated GL261 cells. The values were expressed as the fold change. n = 6 independent samples. **(d)** RT-qPCR for *Ccl2*, *Ccl7*, *Plau*, and *S100a8* in GL261 cells expressing shRNA control (shC) and *Ldha* shRNAs (sh*Ldha*). The values were expressed as the fold change. n = 6 independent samples. **(e, f)** RT-qPCR for *CCL2* and *CCL7* in CT2A cells (**e**) and GSC272 (**f**) treated with or without LDHA recombinant protein (10 ng/ml). n = 6 independent samples. **(g, h)** RT-qPCR for *Ccl2* (**g**) and *Ccl7* (**h**) in shC and sh*Ldha* CT2A cells treated with or without LDHA recombinant protein (10 ng/ml). n = 6 independent samples. **(i)** Representative transwell migration images of THP-1 macrophages following stimulation with recombinant CCL2 and CCL7 proteins (10 ng/ml). Scale bar, 100 μm. **(j-m)** Representative images (**j, l**) and quantification (**k**, **m**) of relative migration of Raw264.7 macrophages from a transwell analysis following stimulation with CM from shC and sh*Ldha* CT2A cells in the presence or absence of CCL2 or CCL7 recombinant proteins (10 ng/ml). n = 5 independent samples. Scale bar, 100 μm. A representative example of five replicates is shown for (I, j, and l). The experiments for (b–d and g-m) were independently repeated at least two times. Data from multiple replicates are presented as mean ± SEM. Statistical analyses were determined by Pearson's correlation test, log-rank test (a), Student’s t-test (b, c, e, f), and one-way ANOVA test (d, g, h, k, m). Source data are provided as a Source Data file.

Supplementary Information Fig. 6

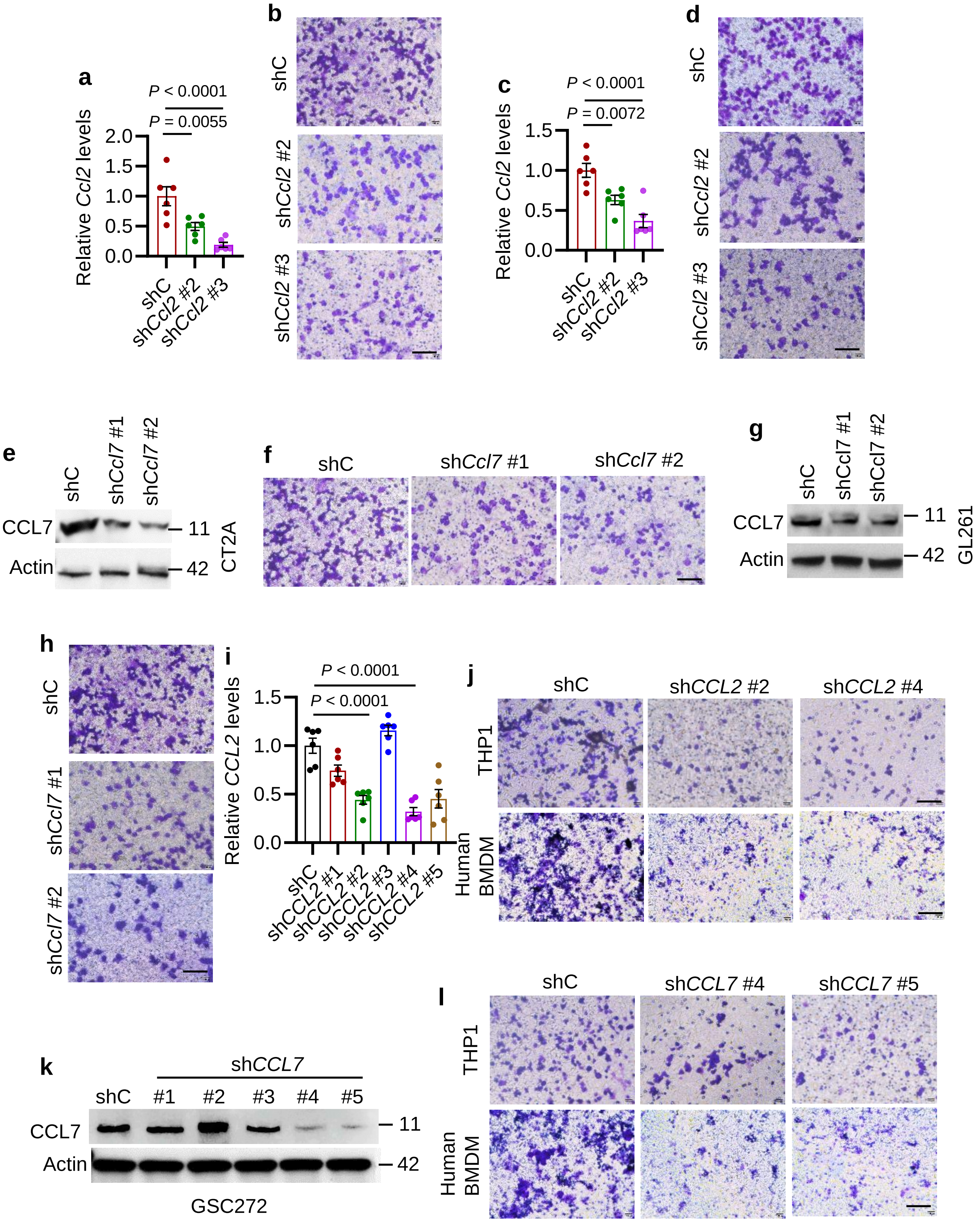

Supplementary Fig. S6. Depletion of glioblastoma cell CCL2 and CCL7 reduces macrophage infiltration. (a) RT-qPCR for *Ccl2* in CT2A cells expressing shC and sh*Ccl2*. The values were expressed as the fold change. n = 6 independent samples. **(b)** Representative transwell migration images of Raw264.7 macrophages following stimulation with conditioned media (CM) from CT2A cells expressing shC and sh*Ccl2*. Scale bar, 100 μm. **(c)** RT-qPCR for *Ccl2* in GL261 cells expressing shC and sh*Ccl2*. The values were expressed as the fold change. n = 6 independent samples. **(d)** Representative transwell migration images of Raw264.7 macrophages following stimulation with CM from GL261 cells expressing shC and sh*Ccl2*. Scale bar, 100 μm. **(e)** Immunoblots of CCL7 in CT2A cells expressing shC and sh*Ccl7*. **(f)** Representative transwell migration images of Raw264.7 macrophages following stimulation with CM from CT2A cells expressing shC and sh*Ccl7*. Scale bar, 100 μm. **(g)** Immunoblots of CCL7 in GL261 cells expressing shC and sh*Ccl7*. **(h)** Representative transwell migration images of Raw264.7 macrophages following stimulation with CM from GL261 cells expressing shC and sh*Ccl7*. Scale bar, 100 μm. **(i)** RT-qPCR for *CCL2* in GSC272 expressing shC and sh*CCL2*. The values were expressed as the fold change. n = 6 independent samples. **(j)** Representative transwell migration images of THP-1 macrophages and human primary BMDMs following stimulation with CM from GSC272 expressing shC and sh*CCL2*. Scale bar, 100 μm. **(k)** Immunoblots of CCL7 in GSC272 expressing shC and sh*CCL7*. **(l)** Representative transwell migration images of THP-1 macrophages and human primary BMDMs following stimulation with CM from GSC272 expressing shC and sh*CCL7.* Scale bar, 100 μm. A representative example of five and three replicates is shown for (b, d, f, h, j, and l) and (e, g, and k), respectively. The experiments were independently repeated at least two to three times. Data from multiple replicates are presented as mean ± SEM. Statistical analyses were determined by one-way ANOVA test (a, c, i). Source data are provided as a Source Data file.

Supplementary Information Fig. 7

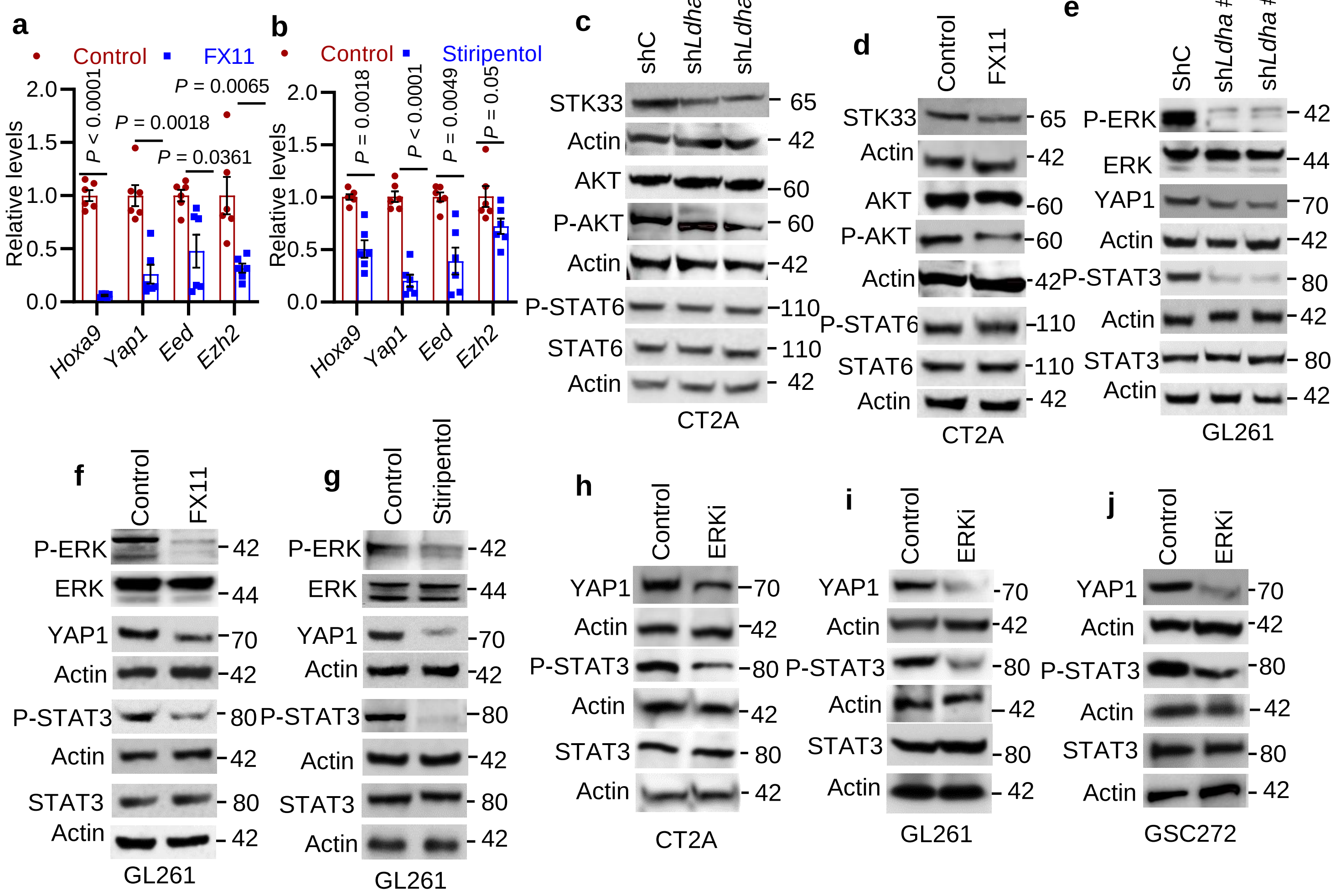

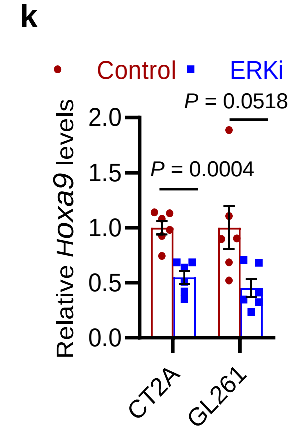

l

Genes	*CCL2*	*CCL7*	Mφ signature
HOXA9	R=0.008<br>P=0.856	R=0.0059<br>P=0.898	R=-0.0135<br>P=0.765
YAP1	R=0.30<br>P<0.0001	R=0.158<br>P=0.0004	R=0.188<br>P<0.0001
TP53	R=0.055<br>P=0.221	R=-0.0028<br>P=0.951	R=-0.031<br>P=0.495
STAT3	R=0.379<br>P<0.0001	R=0.399<br>P<0.0001	R=0.502<br>P<0.0001

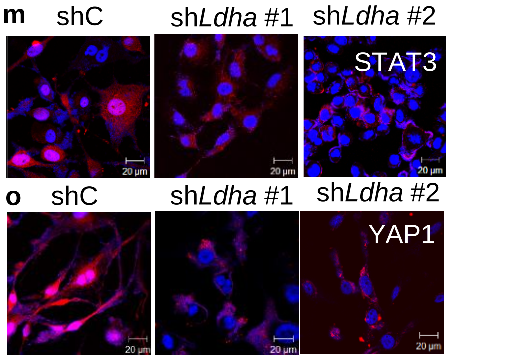

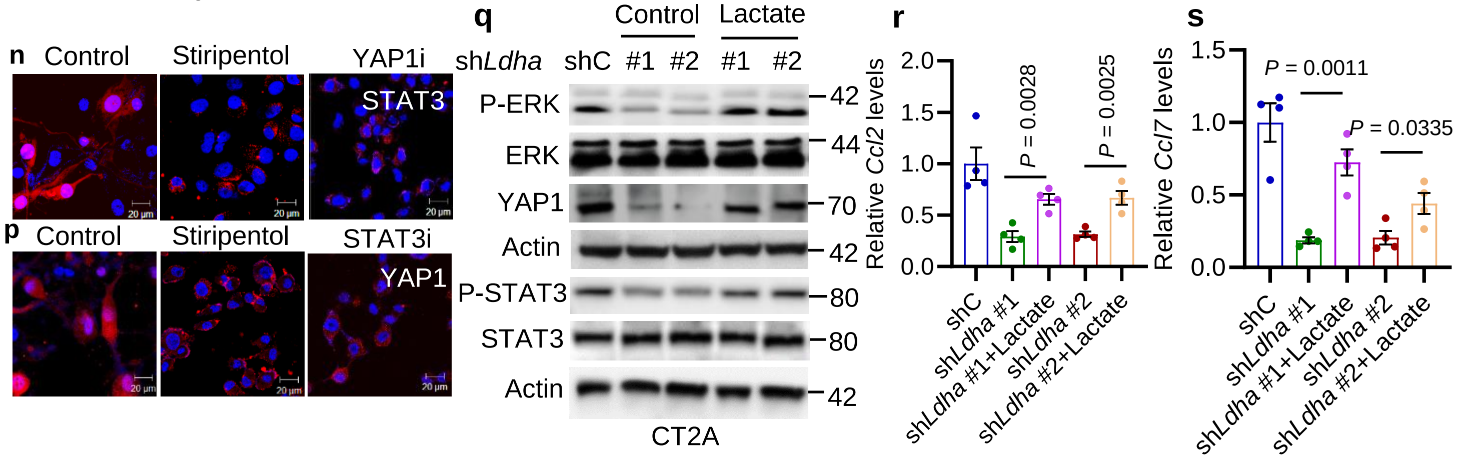

Supplementary Fig. S7. YAP1 and STAT3 regulate LDHA-lactate-ERK axis-induced CCL2 and CCL7 expression. (a) RT-qPCR for *Hoxa9*, *Yap1*, *Eed*, and *Ezh2* in control and FX11-treated GL261 cells. The values were expressed as the fold change. n = 6 independent samples. **(b)** RT-qPCR for *Hoxa9*, *Yap1*, *Eed*, and *Ezh2* in control and stiripentol-treated GL261 cells. The values were expressed as the fold change. n = 6 independent samples. **(c)** Immunoblots of STK33, AKT, P-AKT, STAT6, and P-STAT6 in CT2A cells expressing shRNA control (shC) and *Ldha* shRNAs (sh*Ldha*). **(d)** Immunoblots of STK33, AKT, P-AKT, STAT6, and P-STAT6 in CT2A cells treated with or without FX11 (8 μM). **(e)** Immunoblots of P-ERK, ERK, YAP1, P-STAT3, and STAT3 in GL261 cells expressing shC and sh*Ldha*. **(f, g)** Immunoblots of P-ERK, ERK, YAP1, P-STAT3, and STAT3 in GL261 cells treated with or without FX11 (**f**) or stiripentol (**g**) at 8 and 10 μM, respectively. **(h)** Immunoblots of YAP1, P-STAT3, and STAT3 in CT2A cells treated with or without ERK inhibitor (ERKi) ravoxertinib (1.5 μM). **(i)** Immunoblots of YAP1, P-STAT3, and STAT3 in GL261 cells treated with or without ERKi ravoxertinib (1.5 μM). **(j)** Immunoblots of YAP1, P-STAT3, and STAT3 in GSC272 treated with or without ERKi ravoxertinib (1.5 μM). **(k)** RT-qPCR for *Hoxa9* in control and ERKi ravoxertinib (1.5 μM)-treated CT2A and GL261 cells. The values were expressed as the fold change. n = 6 independent samples. **(l)** The correlation analysis between *HOXA9*, *YAP1*, *TP53*, and *STAT3* with *CCL2*, *CCL7*, and macrophage signature in TCGA glioblastoma dataset (n = 478). *R* and *P* values are shown. Blue colors indicate positive correlation. **(m)** Immunofluorescence for STAT3 in CT2A cells expressing shC or sh*Ldha*. Scale bar, 20 μm. **(n)** Immunofluorescence for STAT3 in CT2A cells treated with or without stiripentol (10 μM) or YAP-TEAD interaction inhibitor (YAP1i) verteporfin (1 μM). Scale bar, 20 μm. **(o)** Immunofluorescence for YAP1 in CT2A cells expressing shC or sh*Ldha*. Scale bar, 20 μm. **(p)** Immunofluorescence for YAP1 in CT2A cells treated with or without stiripentol (10 μM) or STAT3 inhibitor (STAT3i) WP1066 (10 μM). Scale bar, 20 μm. **(q)** Immunoblots of P-ERK, ERK, YAP1, P-STAT3, STAT3, and actin in LDHA-depleted (sh*Ldha*) CT2A cells treated with or without lactate (1 mM). (**r, s**) RT-qPCR for *Ccl2* (**r**) and *Ccl7* (**s**) in CT2A cells expressing shC and sh*Ldha*) treated with or without lactate (1 mM). n = 4 independent samples. A representative example of three replicates is shown for (c-j and m-q). The experiments were independently repeated at least two to three times. Data from multiple replicates are presented as mean ± SEM. Statistical analyses were determined by Student's t-test (a, b, k, r, s) and Pearson's correlation test (l). Source data are provided as a Source Data file.

Supplementary Information Fig. 8

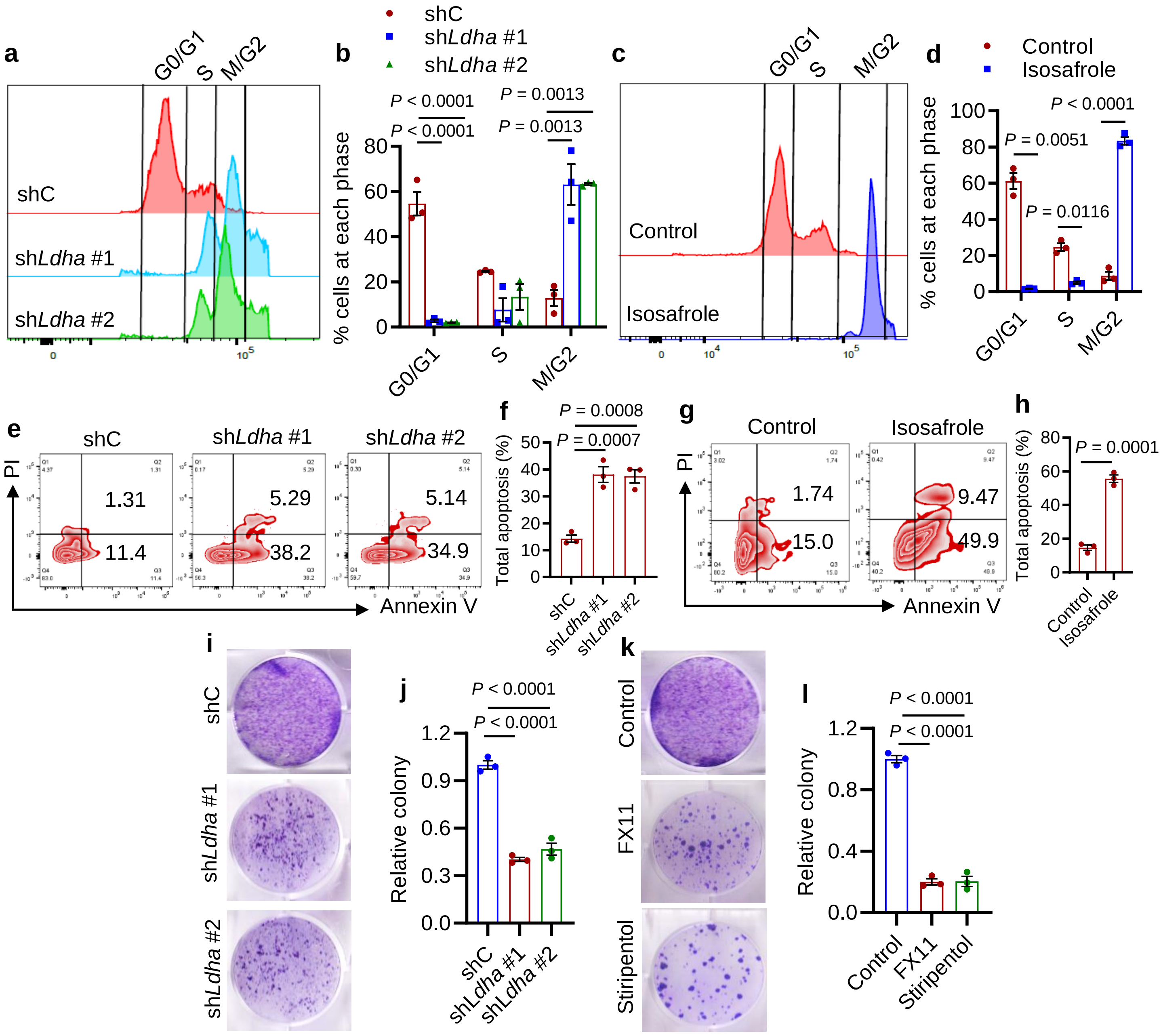

Supplementary Fig. S8. Inhibition of glioblastoma cell LDHA affects cell cycle, apoptosis, and proliferation. (a, b) Representative images (**a**) and quantification (**b**) of flow cytometry cell cycle analysis in CT2A cells expressing shRNA control (shC) and *Ldha* shRNAs (sh*Ldha*). n = 3 independent samples. **(c, d)** Representative images (**c**) and quantification (**d**) of flow cytometry cell cycle analysis in CT2A cells treated with or without isosafrole (10 μM). n = 3 independent samples. **(e, f)** Representative images (**e**) and quantification (**f**) of flow cytometry apoptosis analysis in CT2A cells expressing shC and sh*Ldha*. n = 3 independent samples. **(g, h)** Representative images (**g**) and quantification (**h**) of flow cytometry apoptosis analysis in CT2A cells treated with or without isosafrole (10 μM). n = 3 independent samples. **(i, j)** Representative images (**i**) and quantification (**j**) of colony formation in CT2A cells expressing shC and sh*Ldha*. n = 3 independent samples. **(k, l)** Representative images (**k**) and quantification (**l**) of colony formation in CT2A cells treated with or without FX11 (8 μM) or stiripentol (10 μM). n = 3 independent samples. The experiments were independently repeated at least two times. Data from multiple replicates are presented as mean ± SEM. Statistical analyses were determined by one-way ANOVA test (b, f, j, l) and Student's t-test (d, h). Source data are provided as a Source Data file.

Supplementary Information Fig. 9

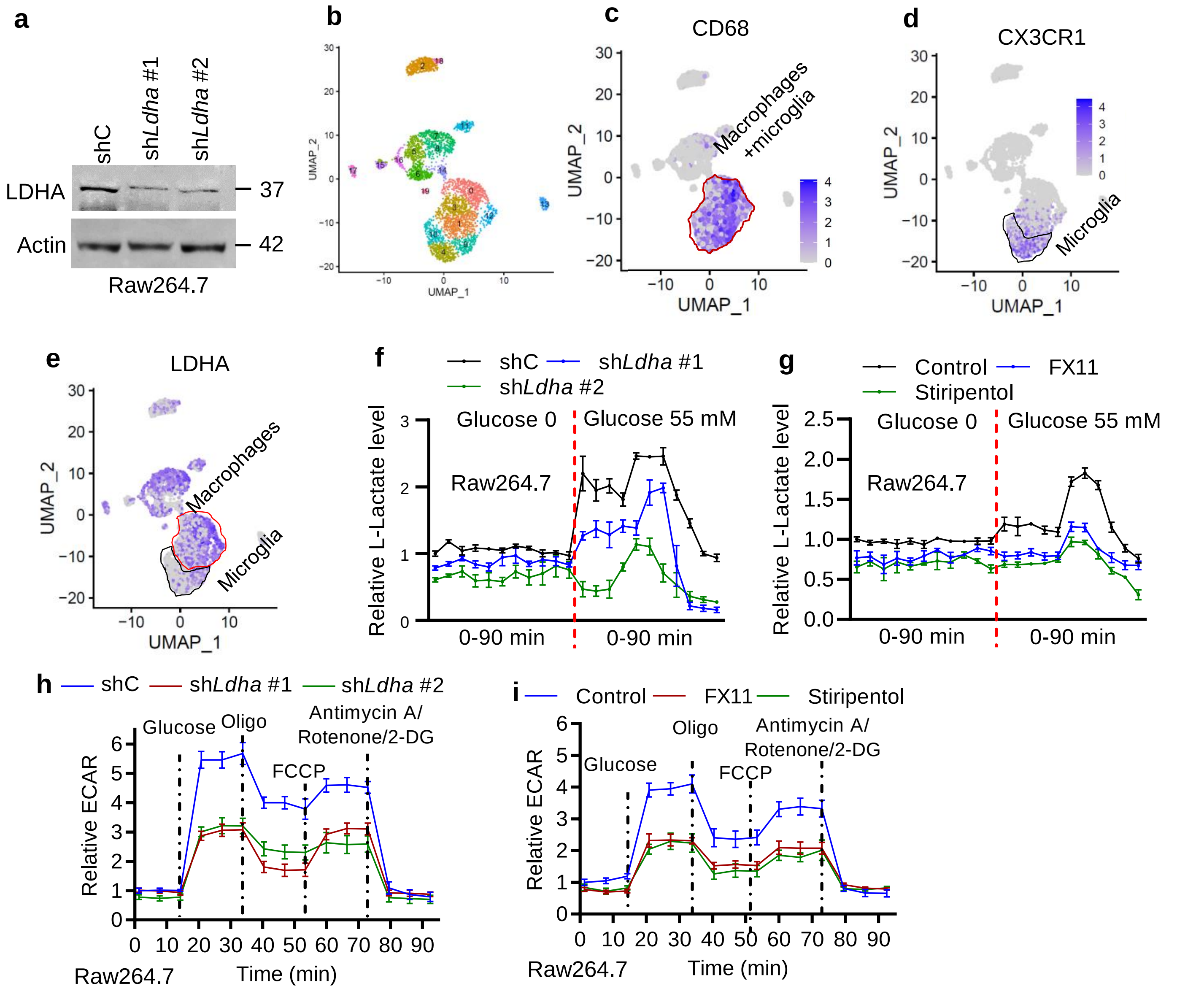

Supplementary Fig. S9. LDHA is expressed in glioblastoma cells and macrophages, and inhibition of LDHA reduces glycolytic activity in macrophages. (a) Immunoblots of LDHAi cell lysates of Raw264.7 macrophages expressing shRNA control (shC) and *Ldha* shRNAs (sh*Ldha*). A representative example of three replicates. **(b)** UMAP dimensional reduction of single cells from tumor samples of a cohort of four glioblastoma patients [41]. **(c)** UMAP dimensional reduction of macrophage and microglia (as highlighted) on the basis of *CD68* expression pattern. **(d)** UMAP dimensional reduction of microglia (as highlighted) on the basis of *CX3CR1* expression pattern. **(e)** Gene expression pattern representing single-cell gene expression of *LDHA* in macrophages, microglia (as highlighted), and other glioblastoma cells. Intensity of the blue color indicates the expression of individual cells. **(f)** Glycolytic activity (lactate level) assay on Raw264.7 macrophages expressing shC and sh*Ldha* in the presence or absence of glucose (55 mM). n = 3 independent samples. **(g)** Glycolytic activity (lactate level) assay on Raw264.7 macrophages treated with or without LDHA inhibitor FX11 (8 μM) or stiripentol (10 μM) in the presence or absence of glucose (55 mM). n = 3 independent samples. **(h)** Extracellular acidification rate (ECAR) of Raw264.7 macrophages expressing shC and sh*Ldha*. ECAR was obtained from the Seahorse experiments and glucose was added at indicated time point. n = 6 independent samples. **(i)** ECAR of Raw264.7 macrophages treated with or without LDHA inhibitor FX11 (8 μM) or stiripentol (10 μM). n = 6 independent samples. ECAR was obtained from the Seahorse experiments and glucose was added at indicated time point. The experiments for (a, h, and i) were independently repeated at least three times. Data from multiple replicates are presented as mean ± SEM. Source data are provided as a Source Data file.

Supplementary Information Fig. 10

a

	Mφ EVs	CT2A EMφ EVs	GL261 EMφ EVs
Size (nm)	149.4 ± 1.9	173.8 ± 3.3	172.9 ± 2.1 nm
Concentration (particles/ml)	2.57E+08 ± 1.39E+07	2.62E+08 ± 1.14E+06	2.44E+08 ± 1.27E+66

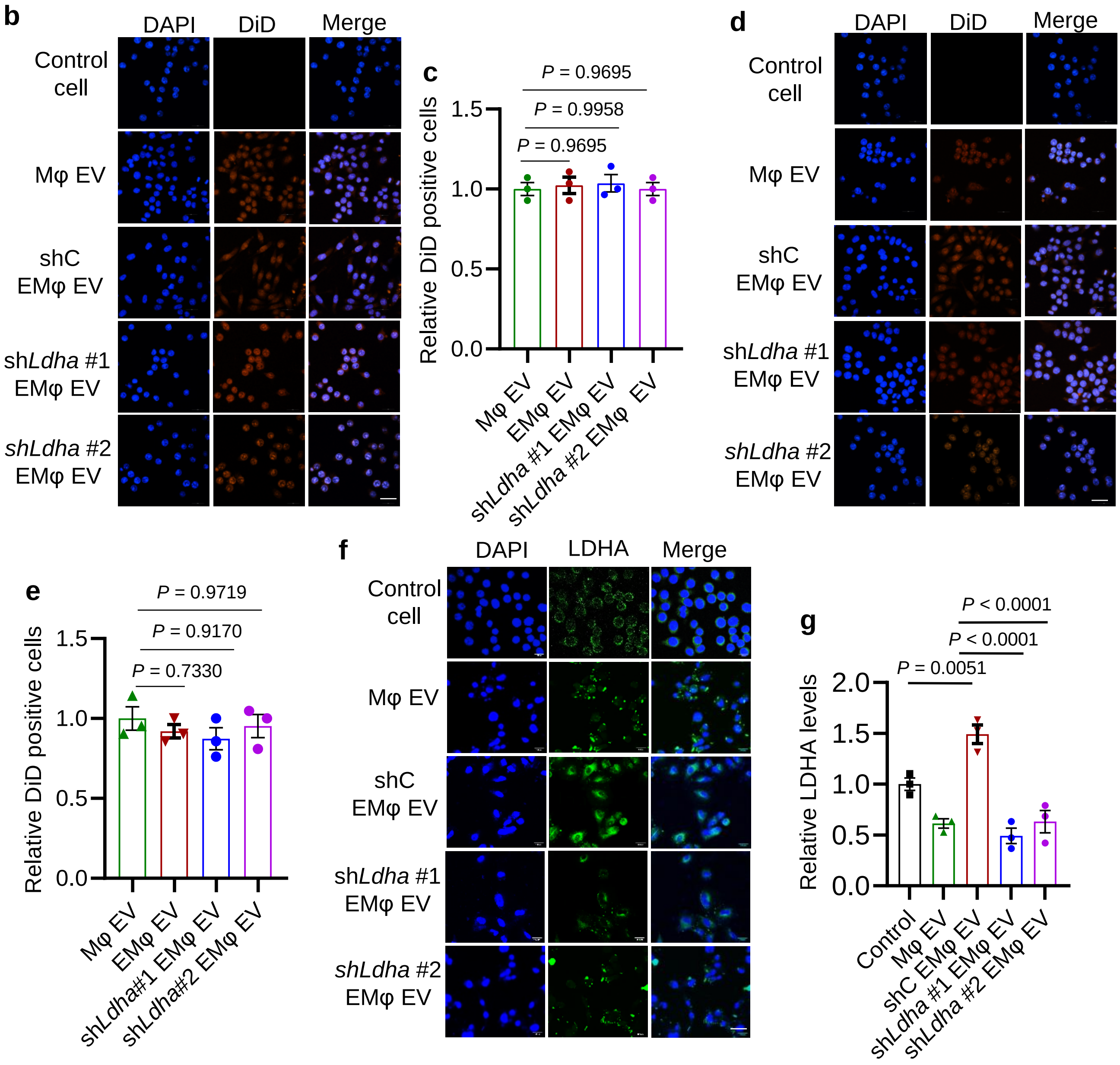

Supplementary Fig. S10. TAM-derived EVs deliver LDHA from macrophages to glioblastoma cells. (a) Quantification of average diameter and concentration of extracellular vesicles (EVs) isolated from control Raw264.7 macrophages and CT2A/GL261 conditioned media-educated macrophages (EMφ) based on nanoparticle tracking analysis. n = 3 independent samples. **(b, c)** Representative (**b**) and quantification (**c**) of DiD positive cells out of total CT2A cells incubated with DiD-labeled EVs (500 ng) isolated from Raw264.7 Mφ and EMφ expressing shRNA control (shC) and *Ldha* shRNAs (*shLdha*) for 24 hrs. Scale bar, 200 μm. n = 3 independent samples. **(d, e)** Representative (**d**) and quantification (**e**) of DiD positive cells out of total GL261 cells incubated with DiD-labeled EVs (500 ng) isolated from Raw264.7 Mφ and EMφ expressing shC and *shLdha* for 24 hrs. Scale bar, 200 μm. n = 3 independent samples. **(f, g)** Representative images (**f**) and quantification (**g**) of immunofluorescence for LDHA in GL261 cells incubated with EVs (500 ng) isolated from control Mφ, GL261 EMφ expressing shC or sh*Ldha* for 24 hrs. Scale bar, 200 μm. n = 3 independent samples. The experiments were independently repeated at least two to three times. Data from multiple replicates are presented as mean ± SEM. Statistical analyses were determined by one-way ANOVA test (a, c, e, g). Source data are provided as a Source Data file.

Supplementary Information Fig. 11

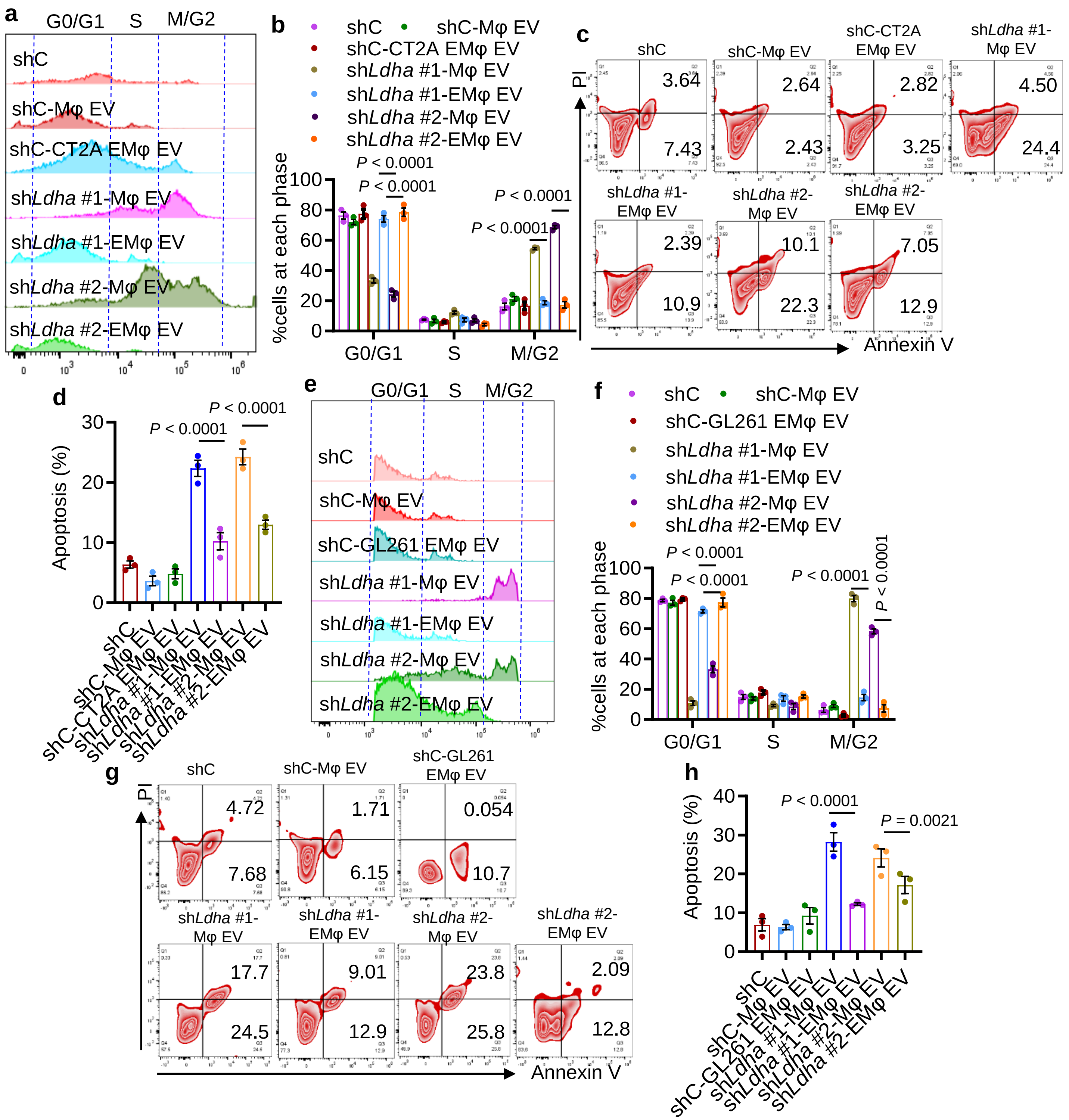

Supplementary Fig. S11. TAM-derived EVs promote glioblastoma cell growth via LDHA. (a, b) Representative (**a**) and quantification (**b**) of flow cytometry cell cycle analysis of shC and sh*Ldha* CT2A cells treated with EVs (500 ng) isolated from Raw264.7 macrophages and CT2A EMφ. n = 3 independent samples. **(c, d)** Representative (**c**) and quantification **(d)** of flow cytometry apoptosis analysis in shC and sh*Ldha* CT2A cells treated with EVs (500 ng) isolated from Raw264.7 Mφ and CT2A EMφ. n = 3 independent samples. **(e, f)** Representative (**e**) and quantification **(f)** of flow cytometry cell cycle analysis of shC and sh*Ldha* GL261 cells treated with EVs (500 ng) isolated from Raw264.7 Mφ and GL261 EMφ. n = 3 independent samples. **(g, h)** Representative (**g**) and quantification **(h)** of flow cytometry apoptosis analysis in shC and sh*Ldha* GL261 cells treated with EVs (500 ng) isolated from Raw264.7 Mφ and GL261 EMφ. n = 3 independent samples. The experiments were independently repeated at least three times. Data presented as mean ± SEM and were analysed by one-way ANOVA test (b, d, f, h). Source data are provided as a Source Data file.

Supplementary Information Fig. 12

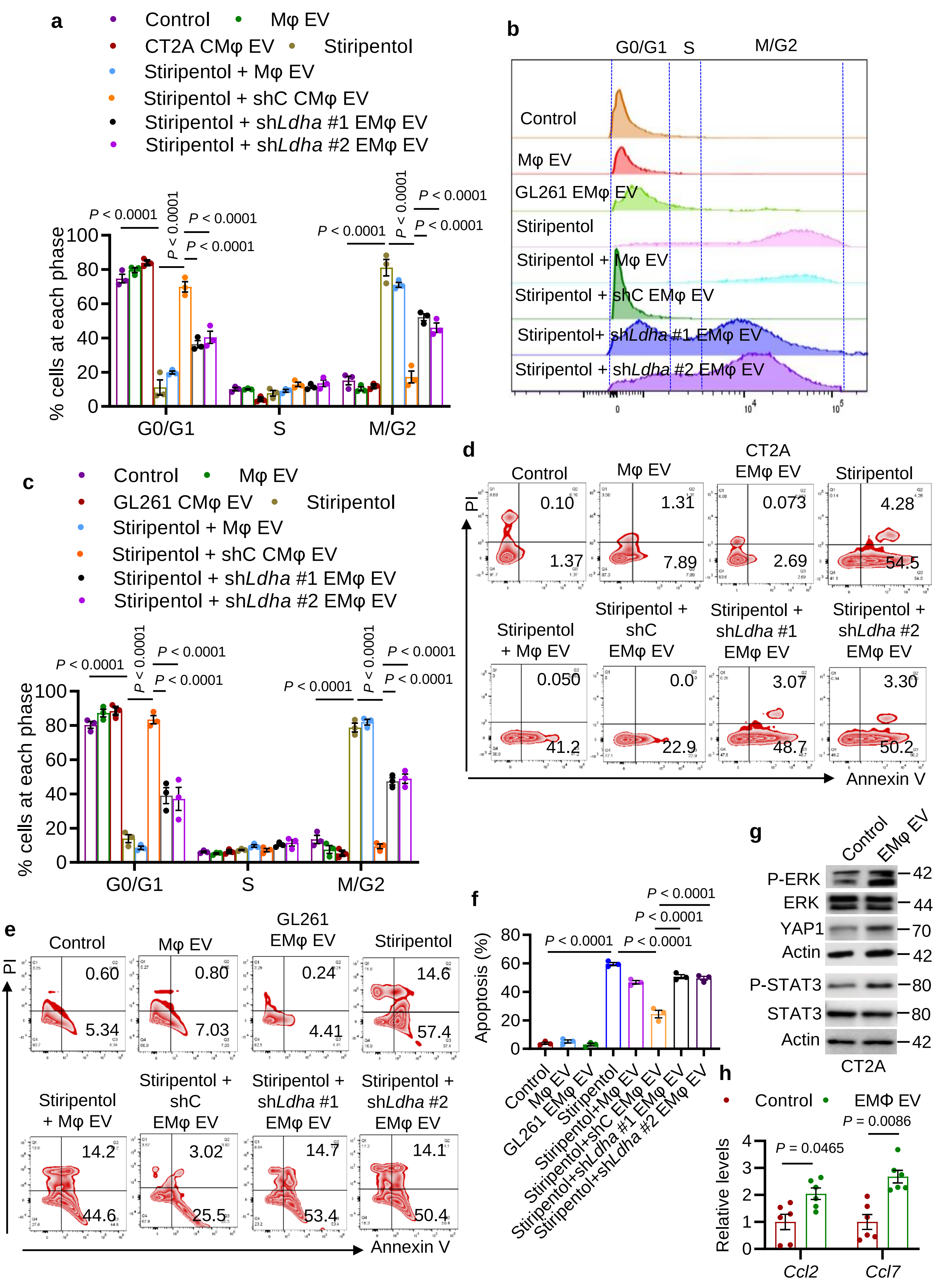

Supplementary Fig. S12. Depletion of LDHA in macrophages impairs the pro-tumor effect of TAM-derived EVs. (a) Quantification of flow cytometry cell cycle analysis of CT2A cells treated with extracellular vesicles (EVs, 500 ng) isolated from Raw264.7 macrophages (Mφ) and CT2A conditioned media-educated macrophages (EMφ), or treated with stiripentol (10 μM) in the presence or absence of EVs isolated from CT2A EMφ expressing shRNA control (shC) and *Ldha* shRNAs (sh*Ldha*). n = 3 independent samples. **(b, c)** Representative images (**b**) and quantification (**c**) of flow cytometry cell cycle analysis of GL261 cells treated with EVs (500 ng) isolated from Raw264.7 Mφ and GL261EMφ, or treated with stiripentol (10 μM) in the presence or absence of EVs isolated from GL261 EMφ expressing shC and sh*Ldha*. n = 3 independent samples. **(d)** Representative images of flow cytometry apoptosis analysis in CT2A cells treated with EVs (500 ng) isolated from Raw264.7 Mφ and CT2A EMφ, or treated with stiripentol (10 μM) in the presence or absence of EVs isolated from CT2A EMφ expressing shC and sh*Ldha*. **(e, f)** Representative images (**e**) and quantification (**f**) of flow cytometry apoptosis analysis in GL261 cells treated with EVs (500 ng) isolated from Raw264.7 Mφ and GL261 EMφ, or treated with stiripentol (10 μM) in the presence or absence of EVs isolated from GL261 EMφ expressing shC and sh*Ldha*. n = 3 independent samples. (**g**) Immunoblots of P-ERK, ERK, YAP1, P-STAT3, STAT3, and actin in CT2A cells treated with or without EVs (500 ng) isolated from CT2A Emφ. (**h**) RT-qPCR for *Ccl2* and *Ccl7* in CT2A cells treated with or without EVs (500 ng) isolated from CT2A EMφ. n = 6 independent samples. A representative example of five and three replicates is shown for (d) and (g), respectively. The experiments for (a-g) were independently repeated at least three times. Data presented as mean ± SEM. Statistical analyses were determined by one-way ANOVA test (a, c, f, h). Source data are provided as a Source Data file.

Supplementary Information Fig. 13

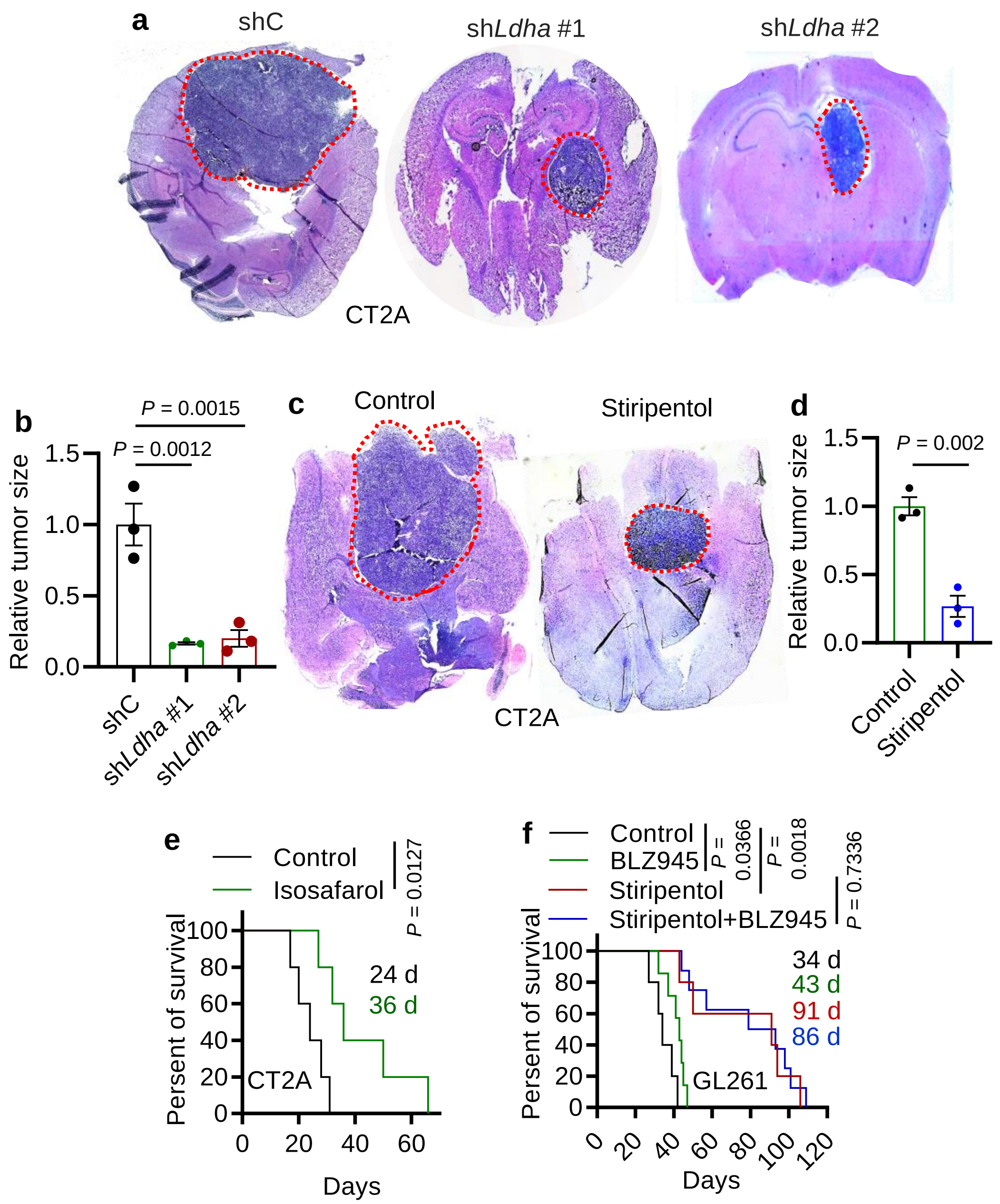

Supplementary Fig. S13. LDHA inhibition reduces glioblastoma growth *in vivo*. (a, b) Representative images (**a**) and quantification (**b**) of H&E staining for CT2A tumors expressing shRNA control (shC) and *Ldha* shRNAs (sh*Ldha*). Tumors were harvested when control mice showing neurologic deficits or moribund. n = 3 independent samples. **(c, d)** Representative images (**c**) and quantification (**d**) of H&E staining for CT2A tumors treated with or without stiripentol (150 mg/kg, i.p., every other day for 6 doses). Tumors were harvested when control mice showing neurologic deficits or moribund. n = 3 independent samples. **(e)** Survival curves of C57BL/6 mice implanted with CT2A (2×10^4 cells). Mice were treated with isosafarol (150 mg/kg, i.p., every other day, 6 doses) beginning at day 8 post-orthotopic injection. (n = 5 mice per group, sharing the control group with Fig. 6c). **(f)** Survival curves of C57BL/6 mice implanted with GL261 cells (2×10^4 cells). Mice were treated with stiripentol (150 mg/kg, i.p., every other day, 6 doses) and BLZ945 (200 mg/kg, oral gavage, every other day, 5 doses) beginning at day 8 post-orthotopic injection. n = 5, 7, 5 and 8 mice for control, BLZ945, stiripentol, and stiripentol + BLZ945 group, respectively. Data presented as mean $\pm$ SEM. Statistical analyses were determined by one-way ANOVA test (b), Student's t-test (d), and log-rank test (e, f). Source data are provided as a Source Data file.

Supplementary Information Fig. 14

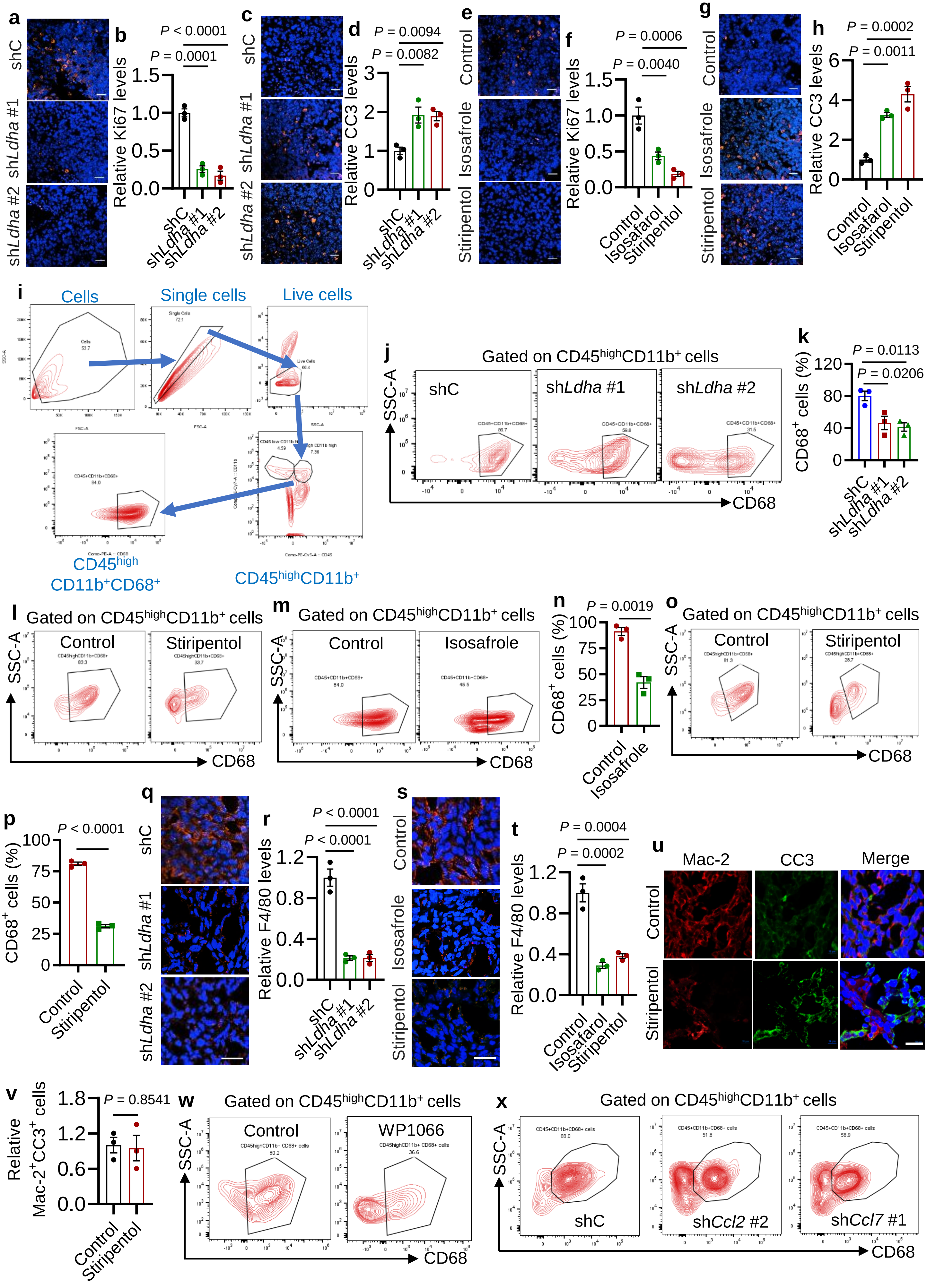

Supplementary Fig. S14. Inhibition of the LDHA-STAT2-CCL2/CCL7 axis reduces glioblastoma cell proliferation and macrophage infiltration in glioblastoma mouse models. (a, b) Immunofluorescence (**a**) and quantification (**b**) of Ki67 positive cells in CT2A tumors expressing shRNA control (shC) and *Ldha* shRNAs (sh*Ldha*). Scale bar, 50 μm. n = 3 independent samples. **(c, d)** Immunofluorescence (**c**) and quantification (**d**) of cleaved caspase 3 (CC3) positive cells in CT2A tumors expressing shC and sh*Ldha*. Scale bar, 50 μm. n = 3 independent samples. **(e, f)** Immunofluorescence (**e**) and quantification (**f**) of Ki67 positive cells in CT2A tumors treated with or without stiripentol and isosafrole. Scale bar, 50 μm. n = 3 independent samples. **(g, h)** Immunofluorescence (**g**) and quantification (**h**) of CC3 positive cells in CT2A tumors treated with or without stiripentol and isosafrole. Scale bar, 50 μm. n = 3 independent samples. **(i)** Flow cytometry gating strategy for intratumoral $CD45^{high}CD11b^{+}CD68^{+}$ macrophages. **(j, k)** Representative (**j**) and quantification (**k**) of flow cytometry analysis for the percentage of $CD68^{+}$ macrophages out of $CD45^{high}CD11b^{+}$ cells in CT2A tumors expressing shC and sh*Ldha*. n = 3 independent samples. **(l)** Representative of flow cytometry analysis for the percentage of $CD68^{+}$ macrophages out of $CD45^{high}CD11b^{+}$ cells in CT2A tumors treated with or without stiripentol.**(m, n)** Representative (**m**) and quantification (**n**) of flow cytometry analysis for the percentage of $CD68^{+}$ macrophages out of $CD45^{high}CD11b^{+}$ cells in CT2A tumors treated with or without isosafrole. n = 3 independent samples. **(o, p)** Representative (**o**) and quantification (**p**) of flow cytometry analysis for the percentage of $CD68^{+}$ macrophages out of $CD45^{high}CD11b^{+}$ cells in 005 GSC tumors treated with or without stiripentol. n = 3 independent samples. **(q, r)** Immunofluorescence (**q**) and quantification (**r**) of F4/80 positive cells in CT2A tumors expressing shC and sh*Ldha*. Scale bar, 50 μm. n = 3 independent samples. **(s, t)** Immunofluorescence (**s**) and quantification (**t**) of F4/80 positive cells in CT2A tumors treated with or without stiripentol and isosafrole. Scale bar, 50 μm. n = 3 independent samples. **(u, v)** Immunofluorescence (**u**) and quantification (**v**) of Mac-2 (macrophage marker) and cleaved caspase 3 (CC3) positive cells in CT2A tumors treated with or without stiripentol. Scale bar, 20 μm. n = 3 independent samples. **(w)** Representative of flow cytometry analysis for the percentage of $CD68^{+}$ macrophages out of $CD45^{high}CD11b^{+}$ cells in CT2A tumors treated with or without WP1066. **(x)** Representative of flow cytometry analysis for the percentage of $CD68^{+}$ macrophages out of $CD45^{high}CD11b^{+}$ cells in shC, sh*Ccl2*, and sh*Ccl7* CT2A tumors. A representative example of three replicates is shown for (l, w, and x). The immunofluorescence experiments were independently repeated at least two to three times. Data presented as mean ± SEM. Statistical analyses were determined by one-way ANOVA test (b, d, f, h, k, r, t) and Student’s t-test (n, p, v). Source data are provided as a Source Data file.

Supplementary Information Fig. 15

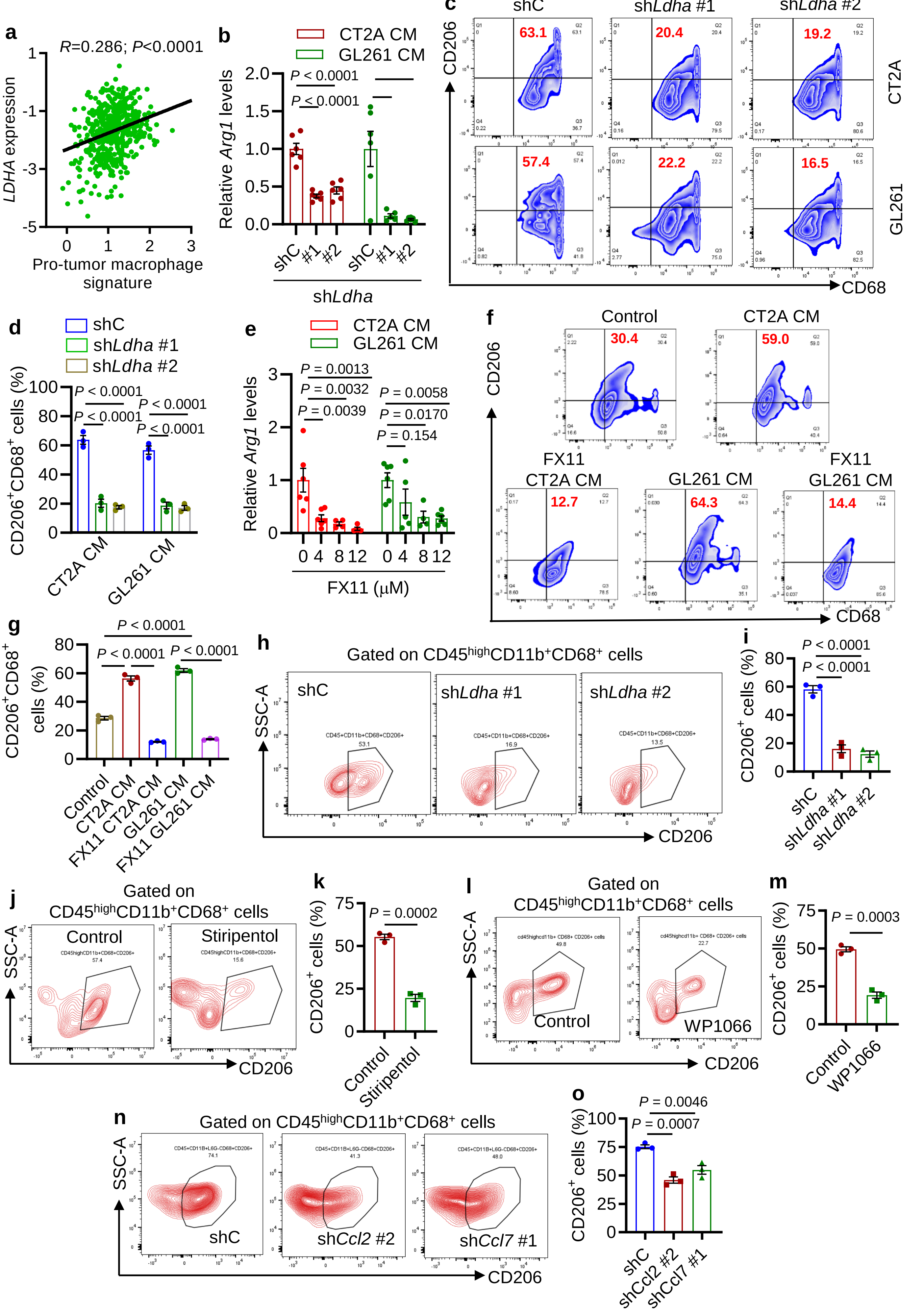

Supplementary Fig. S15. Glioblastoma cell LDHA-YAP1/STAT3-CCL2/CCL7 axis promotes macrophage pro-tumor polarization. (a) The correlation analysis between *LDHA* expression and pro-tumor macrophage signature in TCGA glioblastoma dataset. The pro-tumor macrophage signature was determined by a set of genes as reported previously[32]. *R* and *P* values are shown. (**b**) RT-qPCR for *Arg1* in Raw264.7 macrophages following the treated with or without the conditional media (CM) from CT2A and GL261 cells expressing shRNA control (shC) and *Ldha* shRNAs (sh*Ldha*). n = 6 independent samples. (**c, d**) Representative (**c**) and quantification (**d**) of flow cytometry analysis for the percentage of $CD68^+CD206^+$ cells in Raw264.7 macrophages treated with or without CM from CT2A and GL261 cells expressing shC and sh*Ldha*. n = 3 independent samples. (**e**) RT-qPCR for *Arg1* in Raw264.7 macrophages following the treatment with or without the CM from FX11-treated CT2A and GL261 cells. n = 6 independent samples. (**f, g**) Representative (**f**) and quantification (**g**) of flow cytometry analysis for the percentage of $CD68^+CD206^+$ cells in Raw264.7 macrophages following the treatment with or without the CM from FX11-treated CT2A and GL261 cells. n = 3 independent samples. (**h, i**) Representative (**h**) and quantification (**i**) of flow cytometry analysis for the percentage of $CD206^+$ pro-tumor macrophages out of $CD45^{high}CD11b^+CD68^+$ macrophages in CT2A tumors expressing shC and sh*Ldha*. n = 3 independent samples. (**j, k**) Representative (**j**) and quantification (**k**) of flow cytometry analysis for the percentage of $CD206^+$ pro-tumor macrophages out of $CD45^{high}CD11b^+CD68^+$ macrophages in CT2A tumors treated with or without stiripentol. n = 3 independent samples. (**l, m**) Representative (**l**) and quantification (**m**) of flow cytometry analysis for the percentage of $CD206^+$ pro-tumor macrophages out of $CD45^{high}CD11b^+CD68^+$ macrophages in CT2A tumors treated with or without STAT3 inhibitor WP1066. n = 3 independent samples. (**n, o**) Representative (**n**) and quantification (**o**) of flow cytometry analysis for the percentage of $CD206^+$ pro-tumor macrophages out of $CD45^{high}CD11b^+CD68^+$ macrophages in CT2A tumors expressing shC, sh*Ccl2* and sh*Ccl7*. n = 3 independent samples. The experiments for (b-g) were independently repeated at least two times. Data presented as mean ± SEM. Statistical analyses were determined by one-way ANOVA test (b, d, e, g, i, o) and Student's t-test (k, m). Source data are provided as a Source Data file.

Supplementary Information Fig. 16

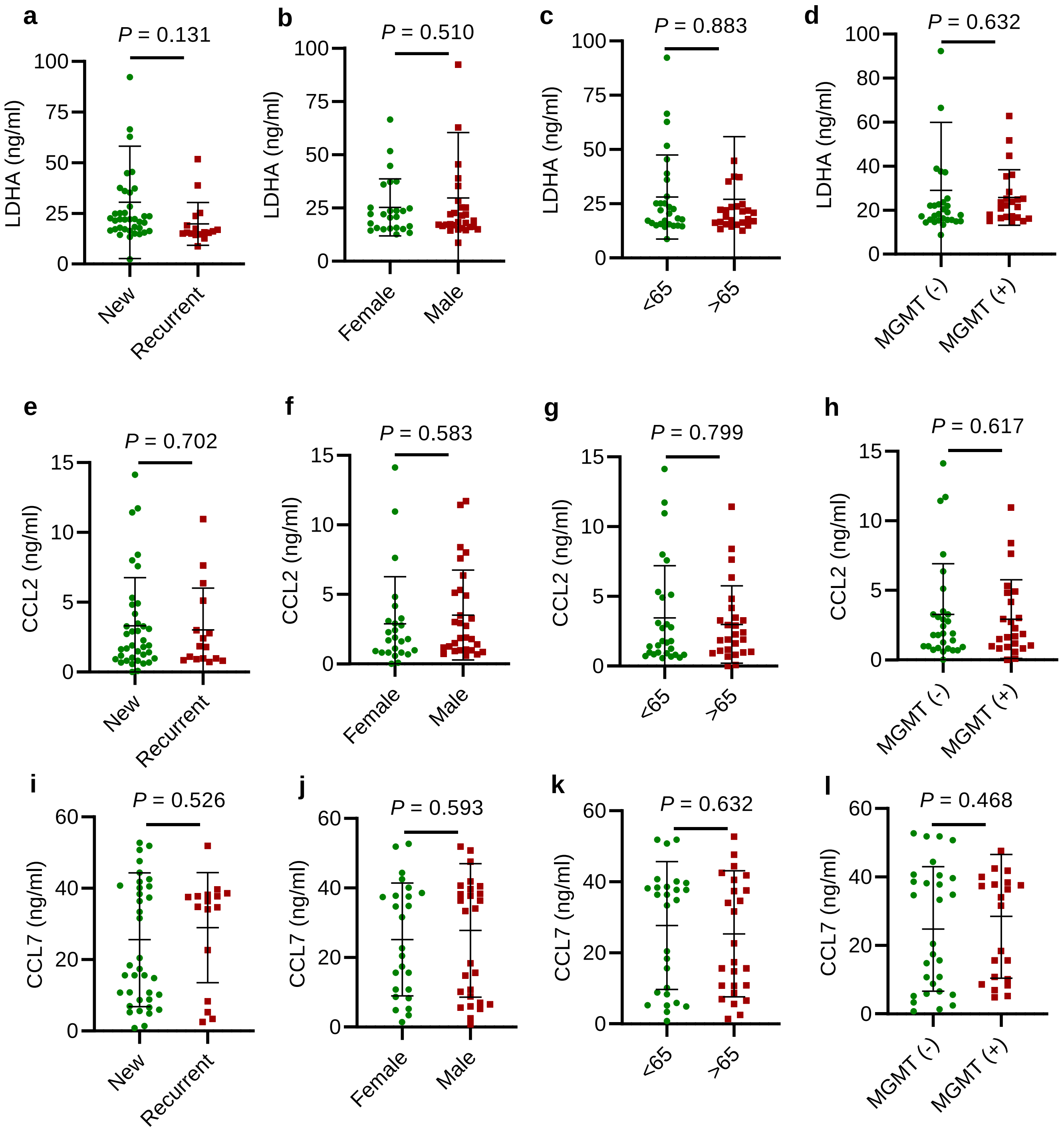

m

	Healthy control	Glioblastoma	*P* value
Size (nm)	164.6 ± 3.8	157.76 ± 2.7	0.6020
Concentration (particles/ml)	4.55E+08 ± 1.81E+06	3.06E+08 ± 7.5E+06	0.8107

n

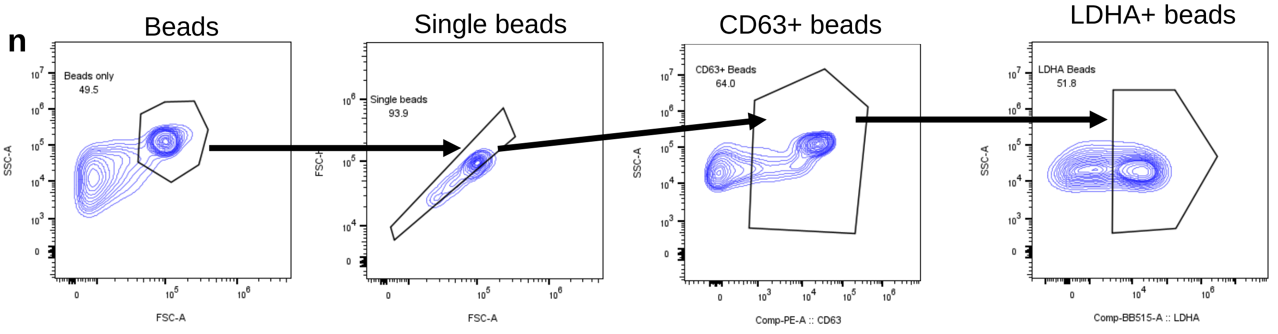

Supplementary Fig. S16. Plasma LDHA, CCL2 and CCL7 levels do not relate to glioblastoma patient recurrence, gender, age and MGMT methylation. (a-d) The level of plasma LDHA from newly diagnosed and recurrent (**a**), female and male (**b**), age <65 and ≥ 65 (**c**), and MGMT unmethylated and methylated (**d**) glioblastoma patients. **(e-h)** The level of plasma CCL2 from newly diagnosed and recurrent (**e**), female and male (**f**), age <65 and ≥ 65 (**g**), and MGMT unmethylated and methylated (**h**) glioblastoma patients. (**i-l**) The level of plasma CCL7 from newly diagnosed and recurrent (**i**), female and male (**j**), age <65 and ≥ 65 (**k**), and MGMT unmethylated and methylated (**l**) glioblastoma patients. **(m)** Quantification of average diameter and concentration of extracellular vesicles **(**EVs) isolated from the plasma of healthy control and glioblastoma patients based on nanoparticle tracking analysis. n = 3 independent samples and the experiments were independently repeated at least three times. **(n)** Gating strategy for examining LDHA level in $CD63^+$ EVs isolated from the plasma of healthy control and glioblastoma patients. Data presented as mean ± SD and analysed by Student's t-test (a-m). Source data are provided as a Source Data file.

Supplementary Information

Table S1. Computational analysis demonstrates a key role of tumor metabolism in regulation of myeloid/leukocyte migration, immune response, and cytokine/chemokine in TCGA GBM patient tumors.

Top ten enriched pathways in metabolism-high patients	Gene Ontology Biological Process	Hallmark pathways	KEGG Pathways
#1	LEUKOCYTE_CHEMOTAXIS	EMT	CYTOKINE_CYTOKINE_RECEPTOR_INTERACTION
#2	CELL_CHEMOTAXIS	TNFA_SIGNALING_VIA_NFKB	FOCAL_ADHESION
#3	LEUKOCYTE_MIGRATION	INFLAMMATORY_RESPONSE	LYSOSOME
#4	MYELOID_LEUKOCYTE_MIGRATION	INTERFERON_GAMMA_RESPONSE	ECM_RECEPTOR_INTERACTION
#5	MYELOID_LEUKOCYTE_ACTIVATION	KRAS_SIGNALING_UP	CHEMOKINE_SIGNALING_PATHWAY
#6	GRANULOCYTE_MIGRATION	IL6_JAK_STAT3_SIGNALING	NOD_LIKE_RECEPTOR_SIGNALING_PATHWAY
#7	GRANULOCYTE_CHEMOTAXIS	INTERFERON_ALPHA_RESPONSE	LEISHMANIA_INFECTION
#8	NEUTROPHIL_MIGRATION	IL2_STAT5_SIGNALING	CELL_ADHESION_MOLECULES_CAMS
#9	NEUTROPHIL_CHEMOTAXIS	COMPLEMENT	COMPLEMENT_AND_COAGULATION_CASCADES
#10	INTERLEUKIN_6_PRODUCTION	HYPOXIA	TOLL_LIKE_RECEPTOR_SIGNALING_PATHWAY

Table S2. Compound library information.

Product Name	Cat. No.	CAS No.	M.Wt	Target	Formula	Clinical Information
D-Cycloserine	HY-B0030	68-41-7	102.09	Antibiotic; Bacterial; iGluR	C3H6N2O2	Launched
Oseltamivir acid	HY-13318	187227-45-8	284.35	Drug Metabolite; Influenza Virus	C14H24N2O4	Launched
Emtricitabine	HY-17427	143491-57-0	247.25	Endogenous Metabolite; HIV; Reverse Transcriptase	C8H10FN3O3S	Launched
Rolipram	HY-16900	61413-54-5	275.34	Bacterial; HIV; Phosphodiesterase (PDE)	C16H21NO3	Phase 2
Lopinavir	HY-14588	192725-17-0	628.8	HIV; HIV Protease; SARS-CoV	C37H48N4O5	Launched
Amprenavir	HY-17430	161814-49-9	505.63	HIV; HIV Protease; SARS-CoV	C25H35N3O6S	Launched
Ofloxacin	HY-B0125	82419-36-1	361.37	Antibiotic; Bacterial; Endogenous Metabolite	C18H20FN3O4	Launched
Necrostatin-1	HY-15760	4311-88-0	259.33	Autophagy; Ferroptosis; Indoleamine 2,3-Dioxygenase (IDO); RIP kinase	C13H13N3OS	No Development Reported

Atorvastatin (hemicalcium salt)	HY-17379	134523-03-8	577.67	Autophagy; Ferroptosis; HMG-CoA Reductase (HMGCR)	C33H34Ca0.5FN2O5	Launched
Oxybenzone	HY-A0067	131-57-7	228.24	Apoptosis; Autophagy; RAR/RXR	C14H12O3	Launched
5-Aminolevulinic acid (hydrochloride)	HY-N0305	9/2/5451	167.59	Apoptosis; Autophagy; Endogenous Metabolite; Mitophagy	C5H10ClNO3	Launched
Lovastatin	HY-N0504	75330-75-5	404.54	Autophagy; Ferroptosis; HMG-CoA Reductase (HMGCR)	C24H36O5	Launched
VER-155008	HY-10941	1134156-31-2	556.4	Autophagy; HSP	C25H23Cl2N7O4	No Development Reported
6-Mercaptopurine	HY-13677	50-44-2	152.18	Autophagy; Endogenous Metabolite; Nucleoside Antimetabolite/Analog	C5H4N4S	Launched
Retinoic acid	HY-14649	302-79-4	300.44	Autophagy; Endogenous Metabolite; PPAR; RAR/RXR	C20H28O2	Launched
Tarenflurbil	HY-10291	51543-40-9	244.26	Autophagy; RAR/RXR	C15H13FO2	Phase 3
Acetazolamide	HY-B0782	59-66-5	222.25	Autophagy; Carbonic Anhydrase	C4H6N4O3S2	Launched
Nortriptyline (hydrochloride)	HY-B1417	894-71-3	299.84	Autophagy; Drug Metabolite	C19H22ClN	Launched
URB-597	HY-10864	546141-08-6	338.4	Autophagy; FAAH; Mitophagy	C20H22N2O3	No Development Reported
Rutin	HY-N0148	153-18-4	610.52	Amyloid-β; Autophagy; Endogenous Metabolite	C27H30O16	Launched
BAY 73-6691	HY-104028	794568-92-6	356.73	Phosphodiesterase (PDE)	C15H12ClF3N4O	No Development Reported
PCC0208009	HY-100771	1668565-74-9	497.63	Indoleamine 2,3-Dioxygenase (IDO)	C29H35N7O	No Development Reported
Vorasidenib	HY-104042	1644545-52-7	414.74	Isocitrate Dehydrogenase (IDH)	C14H13ClF6N6	Phase 3
PF-05085727	HY-102050	1415637-72-7	413.4	Phosphodiesterase (PDE)	C20H18F3N7	No Development Reported
LXR-623	HY-10629	875787-07-8	422.78	LXR	C21H12ClF5N2	Phase 1
IDO1-IN-5	HY-111540	2166616-75-5	396.45	Indoleamine 2,3-Dioxygenase (IDO)	C23H25FN2O3	No Development Reported
IDH889	HY-112289	1429179-07-6	436.48	Isocitrate Dehydrogenase (IDH)	C23H25FN6O2	No Development Reported
IOX4	HY-120110	1154097-71-8	328.33	HIF/HIF Prolyl-Hydroxylase	C15H16N6O3	No Development Reported
ABX-1431	HY-117632	1446817-84-0	507.39	MAGL	C20H22F9N3O2	Phase 2
Balipodect	HY-12472	1238697-26-1	428.42	Phosphodiesterase (PDE)	C23H17FN6O2	Phase 2
SB-3CT	HY-12354	292605-14-2	306.4	MMP	C15H14O3S2	No Development Reported
Nepicastat (hydrochloride)	HY-13289A	170151-24-3	331.81	Dopamine β-hydroxylase	C14H16ClF2N3S	Phase 2
LEI-401	HY-131181	2393840-15-6	421.54	Phospholipase	C24H31N5O2	No Development Reported

DSR-141562	HY-136569	2007975-22-4	414.42	Phosphodiesterase (PDE)	C19H25F3N4O3	No Development Reported
AT-007	HY-129586	2170729-29-8	425.4	Aldose Reductase	C17H10F3N3O3S2	Phase 3
GSK805	HY-12776	1426802-50-7	532.36	ROR	C23H18Cl2F3NO4S	No Development Reported
Apovincaminic acid (hydrochloride salt)	HY-133813A	72296-47-0	358.86	Drug Metabolite	C20H23ClN2O2	No Development Reported
Miquelianin	HY-13930	22688-79-5	478.36	Endogenous Metabolite	C21H18O13	No Development Reported
MDL-28170	HY-18236	88191-84-8	382.45	Proteasome	C22H26N2O4	No Development Reported
Mardepodect	HY-50098	898562-94-2	392.45	Phosphodiesterase (PDE)	C25H20N4O	Phase 2
Mardepodect (hydrochloride)	HY-50098A	2070014-78-5	428.91	Phosphodiesterase (PDE)	C25H21ClN4O	Phase 2
NCT-501	HY-18768	1802088-50-1	416.52	Aldehyde Dehydrogenase (ALDH)	C21H32N6O3	No Development Reported
Vitamin B12	HY-B0315	68-19-9	1355.37	Endogenous Metabolite	C63H88CoN14O14P	Launched
Vardenafil (hydrochloride)	HY-B0442A	224785-91-5	525.06	Endogenous Metabolite; Phosphodiesterase (PDE)	C23H33ClN6O4S	Launched
Chlorzoxazone	HY-B1462	95-25-0	169.57	Cytochrome P450	C7H4ClNO2	Launched
Stiripentol	HY-103392	49763-96-4	234.29	Cytochrome P450	C14H18O3	Launched
Dehydroascorbic acid	HY-110281	490-83-5	174.11	Endogenous Metabolite	C6H6O6	No Development Reported
L-Aspartic acid	HY-N0666	56-84-8	133.1	Endogenous Metabolite	C4H7NO4	No Development Reported
Firibastat	HY-109058	648927-86-0	368.51	Aminopeptidase	C8H20N2O6S4	Phase 3
sn-Glycero-3-phosphocholine	HY-17552	28319-77-9	257.22	AChE; Endogenous Metabolite	C8H20NO6P	Launched
JNJ-1661010	HY-N7062	681136-29-8	365.45	FAAH	C19H19N5OS	No Development Reported
Cyclo(his-pro) (TFA)	HY-101402A	936749-56-3	348.28	Endogenous Metabolite; NF-κB	C13H15F3N4O4	No Development Reported
Progesterone	HY-N0437	57-83-0	314.46	Endogenous Metabolite; Progesterone Receptor	C21H30O2	Launched
Rutin (trihydrate)	HY-W013075	250249-75-3	664.56	Endogenous Metabolite; Others	C27H36O19	Phase 3
Choline (chloride)	HY-B1337	67-48-1	139.62	Endogenous Metabolite; Others	C5H14ClNO	Launched

Table S3. Computational analysis demonstrates a key role of tumor glycolysis in regulation of myeloid/leukocyte migration, immune response, and cytokine/chemokine in TCGA GBM patients.

Top ten enriched pathways in metabolism-high patients	Gene Ontology Biological Process	Hallmark pathways	KEGG Pathways
#1	EXTERNAL_ENCAPSULATING_STRUCTURE_ORGANIZATION	EMT	COMPLEMENT_AND_COAGULATION_CASCADES
#2	LEUKOCYTE_MIGRATION	TNFA_SIGNALING_VIA_NFKB	CYTOKINE_CYTOKINE_RECEPTOR_INTERACTION
#3	LEUKOCYTE_CHEMOTAXIS	HYPOXIA	HEMATOPOIETIC_CELL_LINEAGE
#4	CELL_CHEMOTAXIS	INFLAMMATORY_RESPONSE	ECM_RECEPTOR_INTERACTION
#5	ACUTE_INFLAMMATORY_RESPONSE	COAGULATION	FOCAL_ADHESION
#6	HUMORAL_IMMUNE_RESPONSE	IL6_JAK_STAT3_SIGNALING	LYSOSOME
#7	ADAPTIVE_IMMUNE_RESPONSE	ALLOGRAFT_REJECTION	NOD_LIKE_RECEPTOR_SIGNALING_PATHWAY
#8	PLATELET_DEGRANULATION	COMPLEMENT	AMINO_SUGAR_AND_NUCLEOTIDE_SUGAR_METABOLISM
#9	POSITIVE_REGULATION_OF_CELL_ADHESION	APOPTOSIS	LEUKOCYTE_TRANSENDOTHELIAL_MIGRATION
#10	REGULATION_OF_LEUKOCYTE_MIGRATION	INTERFERON_GAMMA_RESPONSE	LEISHMANIA_INFECTION

Table S4. Computational analysis demonstrates a key role of LDHA in regulation of myeloid/leukocyte migration, immune response, and cytokine/chemokine in TCGA GBM patients.

Top ten enriched pathways in metabolism-high patients	Gene Ontology Biological Process	Hallmark pathways	KEGG Pathways
#1	EXTERNAL_ENCAPSULATING_STRUCTURE	EMT	FOCAL_ADHESION
#2	COLLAGEN_FIBRIL_ORGANIZATION	INFLAMMATORY_RESPONSE	ECM_RECEPTOR_INTERACTION
#3	HUMORAL_IMMUNE_RESPONSE	TNFA_SIGNALING_VIA_NFKB	CYTOKINE_CYTOKINE_RECEPTOR_INTERACTION
#4	NEUTROPHIL_MIGRATION	HYPOXIA	LYSOSOME
#5	MYELOID_LEUKOCYTE_IMMUNITY	COAGULATION	LEUKOCYTE_TRANSENDOTHELIAL_MIGRATION
#6	MYELOID_LEUKOCYTE_ACTIVATION	APOPTOSIS	NOD_LIKE_RECEPTOR_SIGNALING_PATHWAY
#7	INFLAMMATORY_RESPONSE	UV_RESPONSE_DN	COMPLEMENT_AND_COAGULATION_CASCADES

#8	GRANULOCYTE_MIGRATION	IL6_JAK_STAT3_SIGNALING	LEISHMANIA_INFECTION
#9	COLLAGEN_METABOLIC_PROCESS	GLYCOLYSIS	HEMATOPOIETIC_CELL_LINEAGE
#10	MONOCYTE_CHEMOTAXIS	COMPLEMENT	AMINO_SUGAR_AND_NUCLEOTIDE_SUGAR_METABOLISM

Table S5. Computational analysis demonstrates a key role of CCL2 for myeloid/leukocyte migration in TCGA GBM patients.

Top ten enriched pathways	Gene Ontology Biological Process	Gene Ontology Molecular Function	KEGG Enrichment Analysis
#1	Response to bacterium	Cytokine activity	Cytokine-cytokine receptor interaction
#2	Leukocyte migration	Cytokine receptor binding	NOD-like receptor signaling pathway
#3	Regulation of response to wounding	Glycosaminoglycan binding	TNF signaling pathway
#4	Response to molecule of bacterial origin	Peptidase regulator activity	Viral protein interaction with cytokine and cytokine receptor
#5	Response to lipopolysaccharide	Endopeptidase regulator activity	Osteoclast differentiation
#6	Cell chemotaxis	Endopeptidase inhibitor activity	Rheumatoid arthritis
#7	Leukocyte chemotaxis	Heparin binding	NF-kappa B signaling pathway
#8	Myeloid leukocyte migration	Growth factor binding	Complement and coagulation cascades
#9	Granulocyte migration	Chemokine receptor binding	IL-17 signaling pathway
#10	Positive regulation of leukocyte migration	Chemokine activity	Malaria

Table S6. Computational analysis demonstrates a key role of CCL7 for myeloid/leukocyte migration in TCGA GBM patients.

Top ten enriched pathways	Gene Ontology Biological Process	Gene Ontology Molecular Function	KEGG Enrichment Analysis
#1	Leukocyte migration	Cytokine activity	Cytokine-cytokine receptor interaction
#2	Response to bacterium	Cytokine receptor binding	Lipid and atherosclerosis
#3	Regulation of response to wounding	Carbohydrate binding	TNF signaling pathway
#4	Cell chemotaxis	Glycosaminoglycan binding	Tuberculosis
#5	Leukocyte chemotaxis	Peptidase regulator activity	Viral protein interaction with cytokine and cytokine receptor
#6	Myeloid leukocyte migration	Cell adhesion molecule binding	Rheumatoid arthritis

#7	Regulation of leukocyte migration	Heparin binding	IL-17 signaling pathway
#8	Positive regulation of leukocyte migration	Chemokine receptor binding	NF-kappa B signaling pathway
#9	Granulocyte migration	Chemokine activity	Complement and coagulation cascades
#10	Granulocyte chemotaxis	CCR chemokine receptor binding	Legionellosis

Table S7. A list of primers used for RT-qPCR and ChIP-PCR analysis.

**Gene name**	**Forward**	**Reverse**
**RT-qPCR primers**		
Actb	GGCTGTATTCCCCTCCATCG	CCAGTTGGTAACAATGCCATGT
Ccl2	TTAAAAACCTGGATCGGAACCAA	GCATTAGCTTCAGATTTACGGGT
CCL2	CAGCCAGATGCAATCAATGCC	TGGAATCCTGAACCCACTTCT
Ccl7	CCACATGCTGCTATGTCAAGA	ACACCGACTACTGGTGATCCT
S100a8	AAATCACCATGCCCTCTACAAG	CCCACTTTTATCACCATCGCAA
Il1rap	GGAGGAGCCCATTAACTTCCG	CCGTGTCATTGAGGAGGGT
Plau	GCGCCTTGGTGGTGAAAAAC	TTGTAGGACACGCATACACCT
Hoxa9	GGCCTTATGGCATTAAACCTGA	ACAAAGTGTGAGTGTCAAGCG
Yap1	TGAGATCCCTGATGATGTACCAC	TGTTGTTGTCTGATCGTTGTGAT
Eed	GGGGAGATACGGTTATTGCAG	TCATAGGTCCATGCACAAGTGTA
Ezh2	AGTGACTTGGATTTTCCAGCAC	AATTCTGTTGTAAGGGCGACC
Arg1	TTGGGTGGATGCTCACACTG	GTACACGATGTCTTTGGCAGA
**YAP1 ChIP-PCR primers**		
Ccl2	CCACTTTCCATCACTTATCCAGG	TGCTCTGAGGCAGCCTTTTA
Ccl7	TAAGTTCCTATTTCCACCTTTGTCT	AGCTCAGTACTAGAGTTTTTGTCTA
**YAP1 ChIP-PCR primers**		
Ccl2	GCAGAGGCAGGCAAATTTCTGA	TGTATAGTCCTGGCTGTCCTGG
Ccl7	GCCACATCCGGCCCAAACTA	TGAGGGCCATCCCAAAGCAT

Table S8. Patient information.

NSTB ID	Age at time of collection	Sex	New diagnosis vs. recurrent diagnosis	Overall survival - days	Ki-67 Histology		**ELISA**	**IHC staining**	**Exosom e isolation**
**GBM**							**GBM**	**GBM**	**GBM**
NU00323	45-54	Male	Recurrent	1086	5%		NU00323	NU00538	NU00538
NU00538	45-54	Male	New	422	30%		NU00538	NU01713	NU01713
NU00655	50-59	Female	New	959	10%		NU00655	NU01793	NU01793
NU00677	70-69	Female	New	311	20%		NU00677	NU01798	NU01798
NU00761	45-54	Male	New	378	20%		NU00761	NU01808	NU01808
NU00764	75-84	Female	New	283	15%		NU00764	NU01853	NU00323
NU00792	75-84	Male	New	54	20%		NU00792	NU01861	NU00677
NU00908	35-44	Male	New	1094	20%		NU00908	NU01903	NU00792
NU00915	75-84	Female	New	31	50%		NU00915	NU01991	NU00908
NU01063	65-74	Female	Recurrent	339	20%		NU01063	NU01994	NU01107
NU01107	60-69	Female	New	401	15%		NU01107	NU02013	
NU01185	70-69	Male	Recurrent		1-2%		NU01185	NU02064	
NU01197	60-69	Female	Recurrent	874	0.1		NU01197	NU02120	
NU01201	80-79	Female	New	434	Not reported		NU01201	NU02156	
NU01226	80-79	Male	New	189	20%		NU01226	NU02203	
NU01232	75-84	Male	New	231	35%		NU01232	NU02411	

NU01247	50-59	Male	New	691	40%		NU01247	NU02446	
NU01276	45-54	Male	Recurrent	673	Not reported		NU01276	NU02541	
NU01293	65-74	Female	Recurrent	1050	30-40%		NU01293	NU02569	
NU01327	55-64	Male	Recurrent	805	Not reported		NU01327	NU02647	
NU01485	<25	Female	Recurrent		80-90%		NU01485	NU01063	
NU01620	70-69	Male	New	381	20%		NU01620	NU01197	
NU01702	50-59	Male	Recurrent	386	2%		NU01702	NU01327	
NU01713	50-59	Male	New	1025	30%		NU01713	NU01485	
NU01743	65-74	Male	New	102	30%		NU01743	NU02209	
NU01761	65-74	Male	Recurrent		30%		NU01761	NU02254	
NU01793	65-74	Female	New		40%		NU01793	NU02326	
NU01798	65-74	Female	New		30%		NU01798	NU02359	
NU01808	35-44	Female	New	470	40-50%		NU01808	NU02576	
NU01853	65-74	Female	New	370	Not reported		NU01853	NU02718	
NU01861	55-64	Male	New	297	60%		NU01861		
NU01903	55-64	Male	New	434	60-70%		NU01903		
NU01967	55-64	Male	New	138	>80%		NU01967		
NU01991	75-84	Male	New		8-10%		NU01991		
NU01994	65-74	Male	New	389	20%		NU01994		
NU02013	55-64	Female	New		0.5		NU02013		
NU02064	65-74	Female	New		0.2		NU02064		
NU02120	55-64	Male	New		0.4		NU02120		
NU02156	75-84	Female	New	233	0.25		NU02156		
NU02203	35-44	Female	New	470	40-50%		NU02203		
NU02209	55-64	Female	Recurrent	675	0.7		NU02209		
NU02254	55-64	Female	Recurrent	746	0.5		NU02254		
NU02299	55-64	Male	New		30%		NU02299		
NU02326	60-69	Male	Recurrent		0.6		NU02326		
NU02337	45-54	Male	New		50%		NU02337		
NU02359	75-84	Female	Recurrent	149	0.4		NU02359		
NU02411	55-64	Female	New	389	0.4		NU02411		
NU02446	55-64	Female	New		0.4		NU02446		
NU02541	65-74	Female	New		0.15		NU02541		
NU02569	70-79	Male	New		0.25		NU02569		
NU02576	50-59	Female	Recurrent		30%		NU02576		
NU02618	75-84	Male	New		25%		NU02618		
NU02647	65-74	Male	New		0.3		NU02647		
NU02718	55-64	Male	Recurrent		30-40%		NU02718		
**Meningioma**							**Meningioma**		
NU01136	55-64	Male	New				NU01136		
NU01254	55-64	Female	New				NU01254		
NU01305	55-64	Female	New				NU01305		
NU01496	65-74	Male	New				NU01496		
NU01500	65-74	Male	New				NU01500		
NU01657	50-59	Female	New				NU01657		

NU01811	60-69	Male	New				NU01811
NU02071	65-74	Female	New				NU02071
NU02143	60-69	Female	New				NU02143
NU02202	60-69	Female	New				NU02202
NU02331	75-84	Male	New				NU02331
NU02520	60-69	Female	New				NU02520
NU02527	50-59	Male	New				NU02527
NU02530	50-59	Male	New				NU02530
NU02707	<25	Female	New				NU02707
