# Supplementary material for: Lactate dehydrogenase A regulates tumor-macrophage symbiosis to promote glioblastoma progression
Source: Nat Commun. 2024 Mar 5;15:1987. doi: 10.1038/s41467-024-46193-z (PMC10914854; doi:10.1038/s41467-024-46193-z)
Supplement: Supplementary file 3 — Reporting Summary [file 41467_2024_46193_MOESM3_ESM.pdf]

nature portfolio

Corresponding author(s): Peiwen Chen

Last updated by author(s): Dec 19, 2023

Reporting Summary

Nature Portfolio wishes to improve the reproducibility of the work that we publish. This form provides structure for consistency and transparency in reporting. For further information on Nature Portfolio policies, see our Editorial Policies and the Editorial Policy Checklist.

Statistics

For all statistical analyses, confirm that the following items are present in the figure legend, table legend, main text, or Methods section.

n/a	Confirmed	
☐	☒	The exact sample size (n) for each experimental group/condition, given as a discrete number and unit of measurement
☐	☒	A statement on whether measurements were taken from distinct samples or whether the same sample was measured repeatedly
☐	☒	The statistical test(s) used AND whether they are one- or two-sided<br>*Only common tests should be described solely by name; describe more complex techniques in the Methods section.*
☒	☐	A description of all covariates tested
☐	☒	A description of any assumptions or corrections, such as tests of normality and adjustment for multiple comparisons
☐	☒	A full description of the statistical parameters including central tendency (e.g. means) or other basic estimates (e.g. regression coefficient) AND variation (e.g. standard deviation) or associated estimates of uncertainty (e.g. confidence intervals)
☐	☒	For null hypothesis testing, the test statistic (e.g. F, t, r) with confidence intervals, effect sizes, degrees of freedom and P value noted<br>*Give P values as exact values whenever suitable.*
☒	☐	For Bayesian analysis, information on the choice of priors and Markov chain Monte Carlo settings
☒	☐	For hierarchical and complex designs, identification of the appropriate level for tests and full reporting of outcomes
☐	☒	Estimates of effect sizes (e.g. Cohen's d, Pearson's r), indicating how they were calculated

Our web collection on statistics for biologists contains articles on many of the points above.

Software and code

Policy information about availability of computer code

Data collection

Western blot bands were imaged under ChemiDoc Touch Imaging System (Bio-Rad) using ImageLab Version 6.1.0 (Bio-Rad).

Flow cytometry data were collected at Robert H. Lurie Comprehensive Cancer Center Flow Cytometry Core Facility using BD FACSymphony A5 Cell Analyzer as specified in MATERIALS AND METHODS.

Optical density (OD) was measured by a Biotek Synergy 2 SL Microplate Reader for enzyme-linked immunoassay (ELISA) and metabolic assays (glycolytic activity and lactate measurement).

Extracellular vesicle grids were viewed under an FEI Tecnai G2 Spirit Twin TEM

Concentrated extracellular vesicles were analyzed using a NanoSight NS3000 device (Nanosight, Malvern).

Immunofluorescence was captured by using Nikon AX/AX R Confocal Microscope System.

qRT-PCR was performed using SYBR Green PCR Master Mix (Bio-Rad, #1725275) in CFX Connect Real-Time PCR Detection System (Bio-Rad).

Seahorse assay performed using Seahorse XFe96 analyzer (Agilent Technologies).

The Hematoxylin and Eosin (H&E) staining images of tissue sections were captured by TissueFAXS Version 6.0.

March 2021

	Immunohistochemistry images were captured by using EVOS Cell Imaging System.<br><br>Schematic working model were created with BioRender.
Data analysis	Flow cytometry data were analyzed using FlowJo v10.8.1 as detailed in MATERIALS AND METHODS section.<br><br>scRNA-seq data were analyzed using R.<br><br>The number of migrated cells in transwell migration assay and immunofluorescence signal was counted and measured using Image J Version 1.53t (NIH, Bethesda, ML).<br><br>Extracellular vesicle particles were analyzed in each field of view, using the nanoparticle tracking analysis (NTA) software (version 2.3, Nanosight).<br><br>ImageJ version 1.53t was used for scoring positive signals for Immunofluorescence and immunohistochemistry images.<br><br>Statistical analysis was conducted using GraphPad Prism 9 (GraphPad Software, USA). In vitro and in vivo measurement data were presented as the means ± SEM or SD. Comparison between two groups was evaluated using Student's t-test, and multiple comparisons among groups were evaluated using a one-way ANOVA test in Tukey's method. The survival analysis for animal models was performed using Log-rank (Mantel-Cox) test. Correlation analysis was performed using the Pearson test to determine the R and P values.<br><br>Seahorse assay were analysed using GraphPad Prism 9 (GraphPad Software, USA).

For manuscripts utilizing custom algorithms or software that are central to the research but not yet described in published literature, software must be made available to editors and reviewers. We strongly encourage code deposition in a community repository (e.g. GitHub). See the Nature Portfolio guidelines for submitting code & software for further information.

Data

Policy information about availability of data

All manuscripts must include a data availability statement. This statement should provide the following information, where applicable:

- Accession codes, unique identifiers, or web links for publicly available datasets
- A description of any restrictions on data availability
- For clinical datasets or third party data, please ensure that the statement adheres to our policy

Single-cell sequencing data of GSE131928 was used for performing GSC unsupervised sub-clustering for macrophages and microglia. https://www.ncbi.nlm.nih.gov/geo/query/acc.cgi?acc=GSE131928.

For bioinformatics analyses of human GBM patient tumor data, we downloaded the microarray gene expression and survival data of TCGA dataset or other available datasets from GlioVis: http://gliovis.bioinfo.cnio.es/. There are no restrictions for data availability.

Human research participants

Policy information about studies involving human research participants and Sex and Gender in Research.

Reporting on sex and gender	Samples from 33 female and 36 male individuals have been used in this study. Patient information is listed in Supplementary Table 8.<br>1 female have been in this study. Human BMDMs, we isolated from bone marrow aspirates of a donor (23-year old). Detailed information is mentioned in the methods section.
Population characteristics	40 patients are under 65; 29 patients are 65 or older. 38 patients are newly diagnosed with GBM; 16 patients are recurrent; 15 patients are newly diagnosed with meningioma. Detailed information is listed in Supplementary Table 8.<br>1 Human BMDMs isolated from bone marrow aspirates of a donor (Female, 23-year old). Detailed information is mentioned in the methods section.
Recruitment	GBM patient peripheral blood plasma samples (n=69) were collected from the Northwestern Central Nervous System Tissue Bank (NSTB). Patient tumor samples (n=30) from surgically-resected GBMs were collected at the NSTB. All patients were diagnosed according to the WHO diagnostic criteria by neuropathologist Dr. Craig Horbinski. Detailed patient information is provided in Supplementary table 8. Control plasma samples (n=10) from healthy human blood were purchased from Solomonpark (#4345), which are commercially available anonymized and de-identified.
Ethics oversight	According to The George Washington University Institutional Review Board and based on the guidelines from the Office of Human Research Protection, the conducted research meets the criteria for exemption #4 (45 CFR 46.101(b) Categories of Exempt Human Subjects Research) and does not constitute human research.

Note that full information on the approval of the study protocol must also be provided in the manuscript.

Field-specific reporting

Please select the one below that is the best fit for your research. If you are not sure, read the appropriate sections before making your selection.

- [x] Life sciences
- [ ] Behavioural & social sciences
- [ ] Ecological, evolutionary & environmental sciences

For a reference copy of the document with all sections, see nature.com/documents/nr-reporting-summary-flat.pdf

Life sciences study design

All studies must disclose on these points even when the disclosure is negative.

Sample size	No statstical method was used to predetermine sample size. Sample size was chosen based on prior experience and prior published studies with similar layout (see detailed information in the Method section with cited references, including<br>1. Chen, P. et al. Circadian Regulator CLOCK Recruits Immune-Suppressive Microglia into the GBM Tumor Microenvironment. Cancer Discovery 10, 371-381 (2020).<br>2. Xuan, W. et al. Circadian Regulator CLOCK Drives Immunosuppression in Glioblastoma. Cancer Immunology Research 10, 770-784 (2022).<br>3. Chen, P. et al. Symbiotic Macrophage-Glioma Cell Interactions Reveal Synthetic Lethality in PTEN-Null Glioma. Cancer Cell 35, 868-884.e866 (2019).<br>4. Pang, et al. Kunitz-type protease inhibitor TFPI2 remodels stemness and immunosuppressive tumor microenvironment in glioblastoma Nature Immunology volume 24, pages 1654–1670 (2023).
Data exclusions	No data were excluded from the analysis. Datasets used for the specific analyses are reported in the Method section.
Replication	The number of replicates for each experiment is indicated in figure legends. Within each experimental group the reproducibility was successful, although a degree of variability was detected due to inter- individual diversity.
Randomization	We did not perform randomization of study participants or samples within each group because not relevant /needed for this study. The intracranial xenograft tumor model was used throughout this study. Briefly, the animals were assigned into different groups after a week of intracranial injection and received treatments. Since no sign can be observed for mice in the first week after intracranial injection and the animals are all female and of the same age, randomization is not performed.
Blinding	Investigators were not blinded to group allocation because not relevant /needed for this study. Data reported for mouse experiments and all other experiments were not subjective but rather based on quantitative analyses.

Reporting for specific materials, systems and methods

We require information from authors about some types of materials, experimental systems and methods used in many studies. Here, indicate whether each material, system or method listed is relevant to your study. If you are not sure if a list item applies to your research, read the appropriate section before selecting a response.

Materials & experimental systems

n/a	Involved in the study	
	x	Antibodies
	x	Eukaryotic cell lines
x		Palaeontology and archaeology
	x	Animals and other organisms
x		Clinical data
x		Dual use research of concern

Methods

n/a	Involved in the study	
x		ChIP-seq
	x	Flow cytometry
x		MRI-based neuroimaging

Antibodies

Antibodies used	PerCP/Cyanine5.5 anti-mouse CD45 Antibody (BioLegend, #103132) https://www.biolegend.com/fr-fr/products/percp-cyanine5-5-anti-mouse-cd45-antibody-4264<br><br>PE/Cyanine7 anti-mouse/human CD11b Antibody (BioLegend, #101216) https://www.biolegend.com/nl-nl/products/pe-cyanine7-anti-mouse-human-cd11b-antibody-19211<br><br>PE Rat Anti-Mouse CD68 (BD Bioscience, #566386) https://www.bdbiosciences.com/en-eu/products/reagents/flow-cytometry-reagents/research-reagents/single-color-antibodies-ruo/pe-rat-anti-mouse-cd68.566386<br><br>Fixable Viability Dye eFluor™ 780 (Invitrogen, #65-0865-14) https://www.thermofisher.com/order/catalog/product/65-0865-14

LDHA Antibody (CST #2012) https://www.cellsignal.com/products/primary-antibodies/ldha-antibody/2012?site-search-type=Products&N=4294956287&Ntt=ldha&fromPage=plp

CD63 Rabbit pAb (ABclonal, #A5271) https://abclonal.com/catalog-antibodies/CD63RabbitpAb/A5271

ALIX / PDCD6IP Rabbit pAb (ABclonal, #A2215) https://abclonal.com/catalog-antibodies/ALIXPDCD6IPRabbitpAb/A2215

Calnexin Antibody pAb (ABclonal, #A15631) https://abclonal.com/catalog-antibodies/CalnexinRabbitpAb/A15631

YAP (D8H1X) XP® Rabbit mAb (CST #14074) https://www.cellsignal.com/products/primary-antibodies/yap-d8h1x-xp-rabbit-mab/14074?_=1668201482489&Ntt=yap&tahead=true

Anti-STAT6 (phospho Y641) antibody (CST #ab28829) https://www.abcam.com/stat6-phospho-y641-antibody-ab28829.html

Stat6 Antibody (CST #9362) https://www.cellsignal.com/products/primary-antibodies/stat6-antibody/9362

Phospho-Stat3 (Tyr705) (D3A7) XP® Rabbit mAb (CST #9145) https://www.cellsignal.com/products/primary-antibodies/phospho-stat3-tyr705-d3a7-xp-rabbit-mab/9145

Stat3 (124H6) Mouse mAb (CST #9139) https://www.cellsignal.com/products/primary-antibodies/stat3-124h6-mouse-mab/9139?_=1668277138123&Ntt=stat3&tahead=true

STK33 (D3S4R) Rabbit mAb (CST #95343) https://www.cellsignal.com/products/primary-antibodies/stk33-d3s4r-rabbit-mab/95343?_=1668277159911&Ntt=STK33&tahead=true

Phospho-Akt (Ser473) (193H12) Rabbit mAb (CST #4058) https://www.cellsignal.com/products/primary-antibodies/phospho-akt-ser473-193h12-rabbit-mab/4058

Akt (pan) (C67E7) Rabbit mAb (CST #4691) https://www.cellsignal.com/products/primary-antibodies/akt-pan-c67e7-rabbit-mab/4691?_=1668277209821&Ntt=AKT&tahead=true

Vinculin (EMD Millipore, #05-386), https://www.emdmillipore.com/US/en/product/Anti-Vinculin-Antibody-clone-V284,MM_NF-05-386

Anti-MAP Kinase 1/2 (Erk1/2) (EMD Millipore #07-467) https://www.emdmillipore.com/Web-US-Site/en_CA/-/USD/ShowDocument-File?ProductSKU=MM_NF-06-182&DocumentId=null&DocumentType=COA&Language=EN&Country=US&ProductBatchNo=2459609&Origin=PDP

p44/42 MAPK (Erk1/2) (137F5) Rabbit mAb (CST #4695) https://www.cellsignal.com/products/primary-antibodies/p44-42-mapk-erk1-2-137f5-rabbit-mab/4695

β-Actin (8H10D10) Mouse mAb (CST #3700) https://www.cellsignal.com/products/primary-antibodies/b-actin-8h10d10-mouse-mab/3700

MCP3 Polyclonal antibody (CCL7) (biorbyt #orb256344) https://www.biorbyt.com/mcp3-antibody-orb256344.html

Anti-mouse IgG, HRP-linked Antibody (CST #7076) https://www.cellsignal.com/products/secondary-antibodies/anti-mouse-igg-hrp-linked-antibody/7076

Anti-rabbit IgG, HRP-linked Antibody (CST #7074) https://www.cellsignal.com/products/secondary-antibodies/anti-rabbit-igg-hrp-linked-antibody/7074

Cleaved Caspase-3 (Asp175) Antibody (CST #9661) https://www.cellsignal.com/products/primary-antibodies/cleaved-caspase-3-asp175-antibody/9661

Ki67/MKI67 Antibody (Novusbio #NB110-89717) https://www.novusbio.com/products/ki67-mki67-antibody_nb110-89717

Biotin anti-mouse/human Mac-2 (Galectin-3) Antibody (Biolegend #125403) https://www.biolegend.com/nl-be/products/biotin-anti-mouse-human-mac-2-galectin-3-antibody-4936

F4/80 antibody (CST, #70076S) https://www.cellsignal.com/products/primary-antibodies/f4-80-d2s9r-xp-rabbit-mab/70076?site-search-type=Products&N=4294956287&Ntt=70076s&fromPage=plp&_requestid=606535

FITC-conjugated annexin V (BioLegend, #640906) https://www.biolegend.com/nl-be/products/fitc-annexin-v-5161

anti-CD16/CD32 cocktail (BioLegend, #103132) https://www.biolegend.com/fr-lu/products/purified-anti-mouse-cd16-32-antibody-190

Validation	All antibodies used in this study were purchased from BD bioscience, Biolegend, Cell Signaling Technology, Abcam, biorbyt, ABclonal and Millipore. All antibodies used are characterized and validated by providers and are suitable for flow-cytometric analyses, western blot, immunofluorescence analysis.<br><br>From BioLegend: Specificity testing of 1-3 target cell types with either single- or multi-color analysis (including positive and negative cell types). Once specificity is confirmed, each new lot must perform with similar intensity to the in-date reference lot. Brightness (MFI) is evaluated from both positive and negative populations. Each lot product is validated by QC testing with a series of titration dilutions. Those listed under "verified" reactivity indicate species that have been tested and confirmed to react in-house by BioLegend.<br><br>From BD Biosciences: The production process underwent stringent testing and validation to assure that it generates a high-quality conjugate with consistent performance and specific binding activity. However, verification testing has not been performed on all conjugate lots. The specificity is confirmed using multiple methodologies that may include a combination of flow cytometry, immunofluorescence, immunohistochemistry or western blot to test staining on a combination of primary cells, cell lines or transfectant models.<br><br>From Cell Signaling Technology: Knock-out validation has been performed on primary antibodies.

Eukaryotic cell lines

Policy information about cell lines and Sex and Gender in Research

Cell line source(s)	detailed in METHODS section.<br><br>The CT2A cell line is derived from a sub-cutaneous, non-metastatic murine glioma.<br><br>The mouse primary bone marrow-derived macrophages (BMDMs) were isolated from C57BL/6 mice and cultured as detailed in MATERIALS AND METHODS section.<br><br>The human primary bone marrow-derived macrophages (BMDMs) were isolated from aspirates of a donor as detailed description in MATERIALS AND METHODS section.<br><br>Human GSC272 were gifted by Dr. Frederick F. Lang from the Brain Tumor Center (The University of Texas MD Anderson Cancer Center).<br><br>Mouse QPP7 and 005 GSCs were provided by Dr. Jian Hu (The University of Texas MD Anderson Cancer Center) and Dr. Samuel D. Rabkin (Massachusetts General Hospital), respectively.<br><br>293T cells were purchased from ATCC (#CRL-3216). 293T is an epithelial-like cell that was isolated from the kidney of a patient.
Authentication	Flow cytometry analyses have been performed to confirm CD68+ primary mouse macrophage cells are CD68+.<br><br>All human cell lines have been validated and authenticated.<br><br>Raw264.7 macrophages,THP-1 macrophages, GL261 cells lines were purchased from the American Type Culture Collection (ATCC).
Mycoplasma contamination	All cell lines are tested negative with mycoplasma contamination.
Commonly misidentified lines<br>(See ICLAC register)	No commonly misidentified cells were used.

Animals and other research organisms

Policy information about studies involving animals; ARRIVE guidelines recommended for reporting animal research, and Sex and Gender in Research

Laboratory animals	C57BL/6 (#0000664), nude mice (#007850), LDHA-flox mice (#030112) and LyzCre mice (#004781) at 5-6 weeks of age were purchased from the Jackson Laboratory. Mice were grouped by 5 animals in large plastic cages and were maintained under pathogen-free conditions. Animals were housed in temperature and humidity controlled rooms with 12:12 light/dark cycles with Teklad LM-485 sterilizable diet.
Wild animals	No wild animals were used in the study.
Reporting on sex	Animals used in this study are female. Sex was not considered as an experimental factor in this study.
Field-collected samples	No field collected samples were used in the study.

Ethics oversight	All animal experiments were performed with the approval of the Institutional Animal Care and Use Committee (IACUC) at Northwestern University.<br>Animals are under protocol IS00017931, IS00016006 and IS00015772.

Note that full information on the approval of the study protocol must also be provided in the manuscript.

Flow Cytometry

Plots

Confirm that:

- [x] The axis labels state the marker and fluorochrome used (e.g. CD4-FITC).
- [x] The axis scales are clearly visible. Include numbers along axes only for bottom left plot of group (a 'group' is an analysis of identical markers).
- [x] All plots are contour plots with outliers or pseudocolor plots.
- [x] A numerical value for number of cells or percentage (with statistics) is provided.

Methodology

Sample preparation	For membrane protein staining, the single-cell suspensions were washed with PBS and then incubated with fixable viability dye (Invitrogen, #5211229035) on ice for 10 min. Following washing with PBS, cells were incubated with the anti-CD16/CD32 cocktail (BioLegend, #103132) in 2% BSA PBS to block Fc receptors for 30 min on ice. Different antibody combinations were added to cell suspensions for 30 min on ice. After washing in PBS, cells were fixed in fixation buffer (BioLegend, #420801) overnight.<br><br>Intracellular protein staining was performed separately or followed by cell surface marker staining. Cells were fixed in fixation buffer at room temperature for 20 min. After washing in permeabilization buffer (0.1% Triton X-100 in PBS) for two times, cells were incubated with antibodies for 20 min at room temperature. Then cell suspensions were rewashed again in permeabilization buffer before testing.<br><br>Cell cycle and apoptosis, flow staining, cells were fixed in ice-cold 70% ethyl alcohol for 30 min at 4 °C. For cell cycle analysis, cells were incubated with RNase A solution (Promega, #A797C; 100 µg/ml) for 5 min at room temperature and then stained with propidium iodide (PI) labeled with RedX (Biolegend, #421301, 50 µg/ml) for 10 min at 4 °C. PI incorporation was analyzed by flow cytometry. For apoptosis analysis, cells were incubated with FITC-conjugated annexin V (BioLegend, #640906) and PI labeled with RedX (1 ug/ml) for 15 min at room temperature and analyzed using a flow cytometer.<br><br>For extracellular vesicle analysis, CD63 exosome capture beads were incubated with primary and second antibodies and then analyzed using a flow cytometer.
Instrument	Samples were read in BD FACSymphony flow cytometer.
Software	Samples were analyzed in FlowJo v10.8.1.
Cell population abundance	N/A. Sorting is not used in this study.
Gating strategy	For mouse brain tumor samples:<br>Macrophages (CD45hiCD11b+CD68+)<br>Activated macrophages (CD45hiCD11b+CD68+CD206+)<br><br>For extracellular vesicle samples:<br>LDHA expression in EVs (CD63+LDHA+)

- [x] Tick this box to confirm that a figure exemplifying the gating strategy is provided in the Supplementary Information.
